# Groups and scores: the decline of cooperation

**DOI:** 10.1098/rsif.2018.0158

**Published:** 2018-07-04

**Authors:** Stefano Duca, Heinrich H. Nax

**Affiliations:** ETH Zürich, D-GESS, 8092 Zürich, Switzerland

**Keywords:** cooperation, public goods games, indirect reciprocity, image score, reputation

## Abstract

Cooperation among unrelated individuals in social-dilemma-type situations is a key topic in social and biological sciences. It has been shown that, without suitable mechanisms, high levels of cooperation/contributions in repeated public goods games are not stable in the long run. Reputation, as a driver of indirect reciprocity, is often proposed as a mechanism that leads to cooperation. A simple and prominent reputation dynamic function through scoring: contributing behaviour increases one's score, non-contributing reduces it. Indeed, many experiments have established that scoring can sustain cooperation in two-player prisoner's dilemmas and donation games. However, these prior studies focused on pairwise interactions, with no experiment studying reputation mechanisms in more general group interactions. In this paper, we focus on groups and scores, proposing and testing several scoring rules that could apply to multi-player prisoners' dilemmas played in groups, which we test in a laboratory experiment. Results are unambiguously negative: we observe a steady decline of cooperation for every tested scoring mechanism. All scoring systems suffer from it in much the same way. We conclude that the positive results obtained by scoring in pairwise interactions do not apply to multi-player prisoner's dilemmas, and that alternative mechanisms are needed.

## 1. Introduction

Social dilemmas are situations where the optimal decision from the perspective of a self-interested individual conflicts with what is optimal for the group collectively. Examples include public goods [1] and common-pool resources situations [2], as modelled using game theory via, for example, prisoner's dilemmas (PD), voluntary contributions games [3,4] or donation games [5]. The common feature of these interactions is that in the absence of a suitable mechanism [6,7] and given insufficient foresight by the players [8,9], the only stable outcome coincides with the socially undesirable one, i.e. absence of cooperation.^1^ The players fail to cooperate and, as a result, are all worse off than in the collective optimum; a phenomenon often referred to as the ‘tragedy of the commons’ [10,11] or the ‘free riding dilemma’ [12].

One of the most important mechanisms that successfully implements cooperation is ‘reciprocity’ [13,14]. Reciprocity is a behaviour whereby people return benefits for benefits (and hostility with hostility) [15]. Thus, cooperation breeds cooperation and may lead to higher pay-offs in the long run, if people resist the momentary benefits of defection (which, instead, breeds more defection and eventually leads to low pay-offs). Commonly, one distinguishes between direct and indirect reciprocity. Direct reciprocity assumes that a player would cooperate with another person expecting him to do the same in return [16]; under indirect reciprocity, instead, a person does not expect the recipient of his help to reciprocate, but he expects that someone else will [5]: ‘the recipients of an act of kindness are more likely to help in turn, even if the person who benefits from their generosity is somebody else’ [17].

A principal driver of indirect reciprocity is reputation [18], therefore considered as a ‘universal currency’ [19]: cooperating, or refusing to do so and choosing to defect, affects not only one's stage-game pay-off but also one's reputation. When interacting again in the future, players will take each others' reputations into account, thus indirectly reciprocating players who have a good reputation (i.e. that have cooperated in the past). This creates incentives to cooperate beyond the momentary temptations of defection, provided the future benefits of cooperation are substantial. As a result, cooperation may emerge in the presence of suitable reputation mechanisms.

Indeed, reputation—via numerous implementations—has been shown to stabilize high levels of cooperative behaviour in controlled experiments involving human subjects [20–22]. However, an important limitation of prior studies has been the focus on pairwise interactions, while in reality most social interactions unfold in groups [23] involving team production [24]. Producing in teams is particularly relevant in present society as interactions increasingly take place online, involving largely impersonal, crowd interactions.

Moving from pairwise interactions to group interactions substantially complicates matters in theory and in practice. In a group interaction, players might not be able to observe the actions undertaken by others individually, thus making it harder to track and update other players' reputations. Other than in a two-person interaction, one can often not infer the others' individual actions from the aggregated outcome. For instance, when playing a public good game, information regarding individual behaviours may not be available, and the only available information may concern the group as a whole.

This raises the following question: how do reputation mechanisms fare in group interactions? More specifically, as a first step towards addressing the question more generally, we shall here investigate one of the best-known and simplest mechanisms for reputation called ‘scoring’. Our analysis of ‘group scoring’ extends the concept of ‘image scoring’ [25,26], as has been studied widely in pairwise interactions. Under image scoring [5,27], each player has a score (starting at 0) as a proxy for his reputation. Whenever a player has the opportunity to cooperate with someone else, his score is updated: if he cooperates, his score is increased by one, if not it is decreased by one. Thus a player's reputation is continuously reassessed based on the past (in the simplest case, based on the previous decision). A seminal theory result [27] is that the strategy to cooperate with anybody with a non-negative image score is evolutionary stable. Crucially, by refusing to cooperate with someone with a low image score a player is decreasing his own score, thus reducing his own probability of receiving cooperation in the future. Hence, not cooperating with a player with a low image score can be interpreted as a form of punishment. Indeed, in practice, numerous behavioural experiments show that image scoring helps stabilize cooperative behaviour in two-player PDs and donation games [26,28–30].

As we extend scoring mechanisms to group interactions more generally, and to multi-player PDs in particular, we increase the degree of freedom regarding the scoring rules that may apply. Real-world group interactions vary with respect to the information that is available, and typically individuals do not observe all actions undertaken by all other individuals, especially in large groups. The relevant scoring mechanism that applies to a specific group interaction therefore depends on how much information is available to players and how much information each reputation rule requires, as processing the available information correctly may become difficult in larger interactions. Indeed, a conjecture [19] for why image scoring is favoured over other reputation dynamics is that (relatively) little information is required to implement it under full feedback [31]. As such, with limited [32] or partially erroneous feedback [33], sufficiently accurate information is key for mechanism success.

When interacting in groups, information becomes coarser and a single subject may thus find it harder to reap the benefits of ‘reputation-building’, and cooperation may therefore unravel. Recent theory has extended ‘scoring’ methods to group interactions [34]. The baseline establishes a positive cooperation result for the case of image scoring in group interactions.^2^ Furthermore, when only information regarding group performance—but not regarding individual players—is available, ‘group scores’ replace image scores: each player's group score summarizes the aggregate cooperativeness of the groups to which he belonged in the past, without any additional information regarding what players did individually. In this case, theory predicts that cooperation cannot be sustained.

In this paper, we provide the first test of this theory in a group setting considering various informational contexts. Hence, as a first step towards addressing this question more generally, we investigate whether different scoring mechanisms can sustain cooperation in experimental multi-player PDs. In particular, we consider a simple and widely used implementation for scoring mechanisms based on ‘Markovian’ scores, that is, scores that depend only on players' actions from the previous period (memory 1). The basic model we consider is an individual-level binary^3^ Markovian ‘image score’, as investigated theoretically in numerous prior studies (e.g. [5,25,35–39]). For such scores, theory predicts that high levels of cooperation can stabilize, and there exists experimental evidence confirming this in the context of pairwise interaction [32,40]. In fact, concerning the role of memory, existing experimental evidence [40] suggests that Markovian memory already leads to high levels of cooperation and that longer memory increases cooperation further. The goal of the present paper is to investigate whether, for the case of the Markovian baseline, the positive results that were obtained for pairwise interactions carry over to group interactions.

For this, we conducted an extensive laboratory experiment. The baseline is to test image scoring. In addition, we test alternative scoring rules that could apply to group interactions including one where players score each other endogenously through votes. The proposed rules differ with respect to how much information regarding past behaviour of their group-mates is required, ranging from no feedback to full feedback.

The experimental results concerning cooperation are negative: for every scoring mechanism, we observe a steady decline in cooperative behaviour. The decay of cooperation is the same under every mechanism and comparable even with the case when no scoring mechanism is implemented at all. We conclude that positive results regarding cooperation deriving from scoring, as were repeatedly observed in two-player interactions, do not generalize to group interactions. Our results confirm the negative theoretical prediction with respect to coarse group scoring but falsify the positive prediction regarding image scoring in groups.

The rest of this paper is structured as follows. Next, we present the experimental procedure, followed by our results. The Material and methods section contains additional details concerning experimental design and statistical analyses.

## 2. Results

Before presenting results, we briefly discuss the structure of the experiment and introduce the different scoring mechanisms that were tested. For further detail concerning the experimental design, we refer the reader to the Material and methods section and the electronic supplementary material.

### 2.1. Experimental procedure

Our experiment involved 192 subjects playing several, repeated multi-player PDs, resulting in a total of 11 520 on whether to cooperate or not. The experiment had 12 sessions involving 16 subjects each; each session consisted of three different treatments, each played for blocks of 20 rounds (phases). In each treatment, subjects were faced with a different scoring mechanism and treatments differed according to which and in which order the following mechanisms were implemented.

### 2.2. Scoring mechanisms

Scoring mechanisms range between image scoring, providing full feedback about other player's actions, and no scoring, providing no feedback at all (a summary of all the scoring mechanisms is provided in table 1):

— *No scoring*. Subjects receive no information at all regarding the past actions of the other players, and therefore it is the treatment with the lowest informational content. *Expectation*: in this implementation of a repeated multi-player PD, we expect a decay of cooperation resulting in low contribution levels, as shown by numerous previous experiments [26,30,31,41] mainly conducted in voluntary contribution games settings.
— *Image scoring*. This is the treatment with the highest informational content of all, equivalent to the case with a binary image score in two-player interactions. Players are told whether their past and future group-mates cooperated in the previous round. *Expectation*: based on previous experiments on donation games [41] and on theoretical results [34], one could expect a stable high level of cooperation.
— *Group scoring*. Scoring proceeds as in image scoring, except that all group members receive the same score based on the number of cooperators in their group. Subjects are given no direct information about individual decisions. *Expectation*: recent theoretical work [34] suggests that a low level of cooperative behaviour is to be expected.
— *Self-scoring*. Players directly assign the score to their fellow players based on feedback regarding own pay-offs and aggregate contributions in their group. This treatment might contain more or less information than group scoring depending on whether players are truthful when assigning the scores. *Expectation*: in this case, the only Nash equilibrium is for nobody to contribute, independently of the assigned ratings.
— *Image self-scoring*. This is a control treatment for self- and image scoring, where scores are exogenously assigned as if all the players were truthful in the self-scoring treatment. The resulting informational content is, in principle, equivalent to image scoring, but provided in a slightly more complicated format.

**Table 1.** Summary of scoring mechanisms: the table summarizes how much information about other players' actions in the previous round was provided to the players. Regardless of the treatment, all subjects were given feedback regarding the profit made during the round (and hence on the number of contributors in their group).

treatment	feedback provided
no scoring	no feedback about other players' actions
image scoring	feedback about individual actions of others
group scoring	feedback about average behaviour in the group
self-scoring	endogenous feedback
image self-scoring	same as image scoring (control for self- and image scoring)

Every round, subjects were randomly reshuffled and rematched into groups of size 4 and provided with scores feedback, in particular of their group-mates, calculated using the current scoring rule. After deciding whether to cooperate or not, subjects received their personal individual pay-off feedback (thus knowing how many people cooperated in their group) and were assigned updated scores.

It is important to note that, by virtue of our design, the score of a subject only reflected his last action, and that scores did not carry over multiple rounds of the game. Our focus is on situations where mechanisms are introduced or where a new mechanism replaces an old one. Hence, subjects in our experiments always initially played a treatment were no feedback about others' actions or scores was given (Initial phase). After that, two different scoring mechanisms were played in succession (scoring phase 1 and scoring phase 2); see table 2 for the combinations of treatments played during the experiment.

**Table 2.** Combinations of treatments played during the experiment: each row details one of the six treatment combinations in the experiment. Each combination was played twice (in two different experimental sessions).

	treatments' combinations
	initial phase 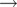 scoring phase 1 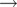 scoring phase 2
	(round 1–20) (round 21–40) (round 41–60)
control treat.	*no scoring 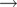 no scoring 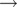 no scoring*
treat. com. 1	*no scoring 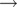 image scoring 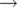 group scoring*
treat. com. 2	*no scoring 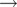 group scoring 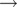 image scoring*
treat. com. 3	*no scoring 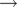 image scoring 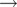 self-scoring*
treat. com. 4	*no scoring 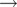 image self-scoring 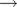 self-scoring*
treat. com. 5	*no scoring 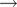 self-scoring 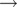 image scoring*

### 2.3. Experimental results

**Figure 1.**
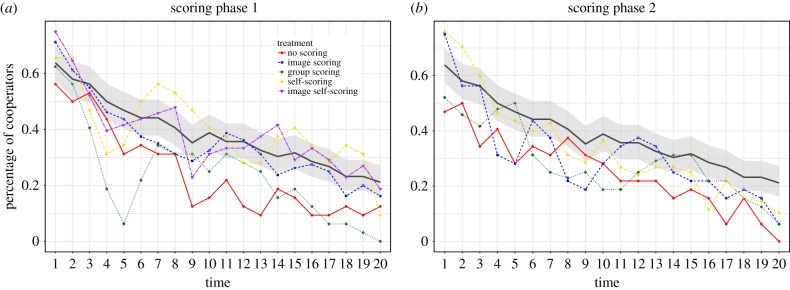
Percentage of cooperation as a function of time for all the treatments: (*a*) and (*b*) the contribution levels observed during the first and second scoring phases of the experiment, respectively. The black line in the background shows the average cooperation observed in the initial phase. As first treatment subjects (i.e. in the initial phase) always played the treatment with no scoring mechanism, it can be treated as a baseline. The grey area represents the binomial proportion confidence interval [42]. The figures show a steady decline in average cooperation. The decline happens in much the same way for all treatments, and independent of the order in which the different treatments were played. (Online version in colour.)

In figure 1, we show the percentage of cooperators as a function of time for all the different treatments.^4^ For all treatments, we observe a steady decline in cooperation; the decay occurs in much the same way, independent of the order in which the different treatments were played.^5^

**Figure 2.**
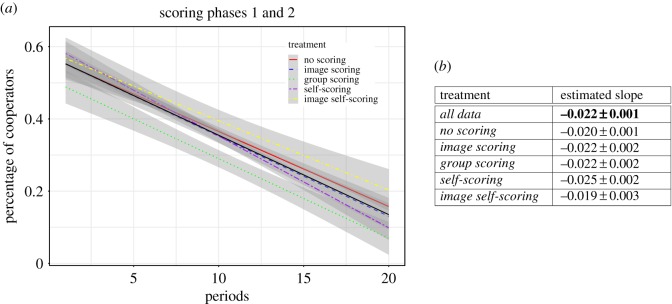
Estimated decays of cooperative behaviour. In figure (*a*), each coloured line illustrates the fitted linear function of a treatment. The grey areas depict the 95% confidence interval. The black line depicts the estimated decay for the entire dataset. Table (*b*) lists the values obtained for the various slope estimators. There is a difference in some of the intercepts of the different lines, but all treatments decline with (statistically) similar slopes. (Online version in colour.)

Even though there are significant statistical differences between some of the observed downward trends (e.g. image scoring is significantly different from no scoring, see table 3), the main difference in treatments can be reduced to a slight offset in the initial percentage of contributors. Figure 2 illustrates that the estimated (linear) decay of cooperation over time occurs at the same speed. Indeed, all the slopes are within the error range of each other. The only notable difference regards the intercept, that is, the initial contributions (figure 2*b*).

**Table 3.** Pairwise Mann–Whitney–Wilcoxon rank sum tests. The table shows the *p*-values obtained from the Mann–Whitney–Wilcoxon Rank Sum test for each pair of treatments. The test was performed only on first-period decisions and excluding the initial phase of every session. Red *p*-values indicate a statistically significant difference (with dark red when *p* < 0.05 and light red when *p* < 0.1) while a black *p*-value indicates no significant difference. (Online version in colour.)

	no scoring	image scoring	group scoring	self-scoring	image self-scoring
no scoring	n.a.	0.063	0.651	0.043	0.029
image scoring	0.063	n.a.	0.158	0.870	0.741
group scoring	0.651	0.158	n.a.	0.115	0.082
self-scoring	0.043	0.870	0.115	n.a.	0.867
image self-scoring	0.029	0.741	0.082	0.867	n.a.

For more details on the statistical analysis, we refer the reader to the Material and methods section.

**Figure 3.**
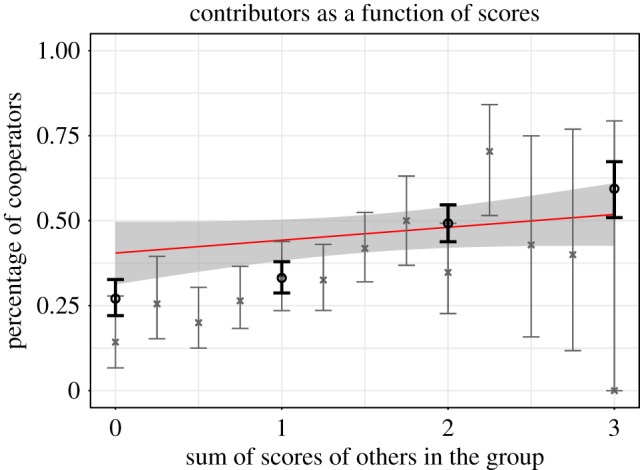
Percentage of cooperators as a function of the observed score in their group. The figure shows how many players contributed to the common pool of their group as a function of the sum of the scores in the group. Black and grey illustrate the image scoring and group scoring cases, respectively. The error bars indicate the 95% binomial proportion confidence interval computed using Wilson score intervals [42]. The continuous red line depicts the percentage of cooperations in the no scoring treatment as a function of what *would have been* the observed score (see the electronic supplementary material for more details); the grey area represents the 95% confidence interval. The figure shows that, even though players cooperate significantly more when the observed sum of scores increases, they do so with a downward bias, when compared with the identity line, especially for high-score values; decisions below the identity line will result in a steady reduction of ‘good players’ in the population, thus lowering the average score and resulting in a spiralling down of cooperative behaviour. (Online version in colour.)

The above results indicate that the scoring mechanisms considered here, even ones that were shown to stabilize high level of cooperation in two-player games (i.e. image scoring), fail to achieve positive results in multi-player interactions. The most plausible explanation is that it is harder to isolate the ‘bad apples’ in a group interaction, resulting in a deterioration of the quality of scores, as perceived by subjects. This kind of imprecision destabilizes cooperation: to keep stable levels of cooperation, players should—on average—cooperate with a frequency at least as high as the observed number of players with a high score in their group, thus maintaining a stable percentage of cooperators in the population. Instead, we observe that, while, ceteris paribus, players do cooperate more with an increased observed score in their group, they do so with a (downward) bias, especially for high sums of scores in the group.^6^ Figure 3 illustrates the case of image and group scoring^7^: in the picture, we can see that players cooperate less than 80% (on average) of what they should cooperate in order to obtain stable cooperation. This behaviour is also confirmed by an analysis of individual decision making: subjects positively react to observed high scores in their group, but they do not ‘reciprocate’ enough for cooperation to be stable. A formal model and analysis of the players' decision making is presented in the electronic supplementary material.

Further contributing to the steady decline of cooperation is the fact that when a high-score player decides not to cooperate because of the presence of low-score subjects in his group, this reduces the score of all his group-mates, not just of the low-score individuals. This results in a steady decay of players with good reputation and cooperative behaviour in the population, and consequentially to a downward spiral of contributions, as observed by Fischbacher *et al.* [47] in a study on (imperfect) conditional cooperation in a public goods experiment.

## 3. Discussion

Scoring methods in general, and image scoring in particular, are simple implementations of reputation mechanisms. They stabilize cooperative behaviour in various standard, two-player social dilemma situations, such as in PD or donation games. Image scoring requires reliable feedback regarding individual-level behaviour. The purpose of this study is to extend such mechanisms to group interactions, in particular to multi-player PD with or without full individual feedback. We propose several scoring rules that could apply in this setting, depending on informational context, and test them in a laboratory experiment. Furthermore, we investigate how an endogenized scoring mechanism could be implemented. The results are unambiguously negative: independent of information, feedback and scoring mechanism, cooperation decays. This includes mechanisms that were previously shown to stabilize cooperation in corresponding two-player cases. A plausible explanation is that individuals cannot be isolated; i.e. defectors cannot be individually punished, and cooperators cannot be individually rewarded. This results in a reaction to the average group score that is increasingly biased towards defection, therefore leading to a steady decrease of high-reputation players in the population that in turn begets lower levels of cooperative behaviour.

On a broader level, our results show that there is still much that we do not know about reputation dynamics. Even though indirect reciprocity is considered one of the main mechanisms through which cooperation can be sustained, there have been very few studies on interactions in group setting. Understanding such settings has become particularly relevant in recent years because, due to the increasing digitalization of our world, more and more interactions take place online where people frequently communicate via crowd platforms and where often explicit reputation tallying is provided as a method to build trust. Owing to the increasing decentralization of interactions, partial or total anonymity of the actors involved can be the norm and reputation is often built on a peer-to-peer basis with members of communities rating each other. For example, a project may involve several groups of individuals, and information on individual level contributions could be imperfectly filtered via several community-layers before reaching the players. With this work, we set up to investigate some of these issues.

A key conclusion is that many positive results on cooperation, as have been observed in pairwise interactions, may not hold anymore when groups are concerned.

There are numerous avenues for future work and many open questions; for example: are dynamics of play in multi-player games fundamentally different from two-player games (as it might be the case for direct reciprocity, see e.g. [48–50])? And if so, could one exploit this to devise a scoring mechanism that is able to sustain higher levels of cooperation? How does group size matter? Could the combination of multiple mechanisms, such as scoring and punishment, lead to higher cooperation? Could the deterioration of the quality of scores be compensated by cumulating the scores over multiple rounds, letting players ‘build’ their reputation? Future work should address such issues and many others, as group structures are an important, ubiquitous aspect of human society.

## 4. Material and methods

### 4.1. The experiment

The experiment was conducted as an experiment on interactive decision-making at the ETH Decision Science Laboratory (DeSciL) in Zurich using the z-Tree [51] software. We ran 12 sessions with 16 participants in each session, for a total of 192 participants. Participants were recruited from the joint subject pool of ETH Zurich and University of Zurich using the hroot [52] sofware and mainly consisted of university students. All procedures adhered to DeSciL's Operational Rules^8^; additional ethics approval was waived following standard DeSciL protocol for members of the laboratory's Review Board. In no way at all does the experiment violate the ethical principles of the Declaration of Helsinki, and subjects were properly incentivized by converting their earnings in real currency with full transparency (i.e. no deception). Each session in the laboratory lasted roughly 1 h during which the players played three treatments for 20 rounds each. On average, subjects earned 33 CHF (roughly 33 USD at the time), with a range of 25 to 40 CHF, including a 5 CHF show-up fee.

First, subjects always played the treatment where no information regarding the past behaviour was provided. After that, subjects played two of the other scoring treatments. The table below details the treatments' combinations.

At the beginning of the session and before each treatment, subjects were given written instructions^9^ explaining what the experiment was about and the game that they were about to play, scoring mechanism included. Before the first treatment, subjects were given some minutes to familiarize with the game with a small training. Before the truthful self-scoring treatment, because of the complexity of the scoring mechanism, subjects also had some minutes to understand how the scoring worked using a score simulator. Screenshots displaying the different treatments can be found in the electronic supplementary material.

As customary, subjects were incentivized by converting their earnings in real currency. Subjects on average earned 33 CHF (roughly 33 USD), including 5 CHF of show-up fee. Earnings ranged from 25 to 40 CHF.

In the following, we define the game that the subjects played in the experiment and the scoring mechanisms that were used.

#### 4.1.1. N-players prisoner's dilemma

The subject played the following game whose aspects were all common knowledge:

1. At the beginning of each round (for 20 rounds), *N* subjects (*N* = |*n*| where *n* ≡ {1, …, 16}) are randomly assigned to four groups of fixed size four.
2. Every subject decides whether to contribute his endowment to the common pool (i.e. whether to cooperate). For player *i*∈*n*, let *c*_*i*_ = 0 and *c*_*i*_ = 1 denote whether player *i* cooperated or not. Starting from the second round of play, players are shown the scores assigned to all players in the previous round. Furthermore, players learn the score of their group-mates in the current round.
3. Subjects receive individual pay-off *ϕ* according to 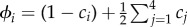.
4. *Scoring*: a score is assigned to each player based on his contribution and depending on the treatment. The score is visible to the other subjects in the following round and it replaces the score from the previous round.

Regardless of the treatment, all subjects were shown the profit that they made during the round and during the entire session; thus each subject was told how many people cooperated in his group in the previous round.

#### 4.1.2. The scoring mechanisms

Depending on treatment, a different score was assigned to each subject. The score was not cumulative over rounds and, every round, subjects were only shown the scores (if any) as were assigned in the previous round. The scoring mechanisms were designed so that the score ranged between 0 and 1 for all treatments.

— *No scoring*. No score was assigned to players during this treatment.
— *Image scoring*. Subjects were assigned a score of 1 if they cooperated in the previous round and 0 if not.
— *Group scoring*. Subjects were assigned a score proportional to how many people in their group contributed to the common pool. The score equalled the number of cooperators in their group divided by the group size (4); thus subjects in the same group all received the same score. More precisely, the score *s*_*i*_ of player *i* in group *G*_*i*_ equals as 
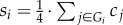
. In principle, the higher subject *i*'s score, the higher is the probability that *i* invested in the group account. If the resulting score is 1 or 0, the group score faultlessly indicates whether a subject cooperated or not, respectively.
— *Self-scoring*. Each subject was asked to rate his/her group awarding a number of stars ranging from 0 to 3. The score of each subject was computed as the sum of all the stars awarded to the group by his group-mates (excluding his own rating) divided by 9 (i.e. the maximum number of stars that a player could be assigned). Therefore, indicating with ⋆_*j*_∈{0, 1, 2, 3} the score assigned by player *j* in group *G*_*i*_ to his group, the score *s*_*i*_ of player *i* in group *G*_*i*_ was computed as 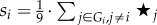. Hence, the score of each subject ranges between 0 (all his group-mates awarded no stars to the group) and 1 (all his group-mates awarded three stars to the group).
— *Image self-scoring*: The score was assigned as in the self-scoring treatment but exogenously. This means that each subject was considered as having awarded a number of stars to his group equal to the number of cooperators (excluding himself) observed in his group. More precisely, for a group *G*_*i*_ we denote with *u*_*i*_∈{0, 1, 2, 3} the sum of the players cooperating in *G*_*i*_ as observed by player *i*; i.e. 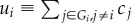. The score *s*_*i*_ of player *i* in group *G*_*i*_ was then computed as 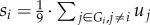. Hence, each score ranges between 0 (all players in that group defected) to 1 (each player in that group cooperated).

### 4.2. Statistical analysis

To determine if treatments significantly differ from one another, we used the Mann–Whitney–Wilcoxon rank sum test [53,54]. Owing to (possible) autocorrelations between same-session decisions, we restricted our analysis to only decisions in the first period. Furthermore, we exclude from the analysis decisions taken during the initial phase. Let *x*^*q*^_*i*_∈{0, 1} denote the decision that player *i* took during the first period of treatment *q*. We obtain 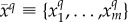 where *m* is the number of players that played treatment *q* (excluding the initial phase). We perform a rank sum test for each pair of treatments: the *p*-value obtained from the test is a measure of how likely it is that 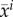 and 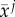 are drawn from the same distribution with the same mode. Table 3 shows the *p*-values for each pair of treatments in the first and second scoring phase of the experiment. A value depicted in red indicates that the two treatments significantly differ from each other.

To obtain figure 2, we performed a linear regression of the contributions to the public good as a function of time for each treatment individually and for all of them combined. An alternative analysis, using a random resampling permutation test, is available in the electronic supplementary material. In the electronic supplementary material, we also provide a model for the decision making of the individual player and fit it to our data. The obtained results are compatible with the ones presented in this manuscript.

## Supplementary Material

SM

## Supplementary Material

Dataset

## Supplementary Material

Instructions

## Endnotes

1 In the remainder of this document, we will use cooperate as a common terminology for related terms like contribute, donate and exert effort

2 I.e. when full information regarding individual decisions is provided.

3 Meaning that the score of a player can only have two values, e.g. 0 and 1.

4 More detailed plots are available in the electronic supplementary material.

5 This decay is in line with similar patterns known from multi-player public goods games (see e.g. [4,43–46]).

6 It is important to note that this effect relies on the players being able to observe the scores in their group. If this is not the case, like in the ‘no scoring’ treatment, no such effect is observed. See the electronic supplementary material for more information.

7 See the electronic supplementary material for the other cases.

8 These implement the standards of behavioural economics including no deception, compatible incentives and payment, minimal earnings, rights to terminate experiments at any time, data anonymity and confidentiality

9 Available in the electronic supplementary material.

## Data accessibility

All collected data are available for download.

## References

[1] Isaac RM, McCue KF, Plott CR 1985 Public goods provision in an experimental environment. J. Public Econ. 26, 51–74. (10.1016/0047-2727(85)90038-6)

[2] Ostrom E 1990 Governing the commons: the evolution of institutions for collective action. Cambridge, UK: Cambridge University Press.

[3] Andreoni J 1988 Why free ride? Strategies and learning in public goods experiments. J. Public Econ. 37, 291–304. (10.1016/0047-2727(88)90043-6)

[4] Isaac RM, Walker JM 1988 Group size effects in public goods provision: the voluntary contributions mechanism. Q. J. Econ. 103, 179–199. (10.2307/1882648)

[5] Nowak MA, Sigmund K 2005 Evolution of indirect reciprocity. Nature 437, 1291–1298. (10.1038/nature04131) 16251955

[6] Riolo RL, Cohen MD, Axelrod R 2001 Evolution of cooperation without reciprocity. Nature 414, 441–443. (10.1038/35106555) 11719803

[7] Hetzer M, Sornette D 2011 A theory of evolution, fairness, and altruistic punishment. PLoS ONE 8, e77041 (10.2139/ssrn.1927919) PMC3834069 24260101

[8] Osborne MJ, Rubinstein A 1994 A course in game theory. Cambridge, MA: MIT Press.

[9] Friedman JW 1971 A non-cooperative equilibrium for supergames. Rev. Econ. Stud. 38, 1–12. (10.2307/2296617)

[10] Ostrom E 1999 Coping with tragedies of the commons. Annu. Rev. Political Sci. 2, 493–535. (10.1146/annurev.polisci.2.1.493)

[11] Hardin G 1968 The tragedy of the commons. Science 162, 1243–1248. (10.1126/science.162.3859.1243) 5699198

[12] Baumol WJ 1952 Welfare economics and the theory of the state. London, UK: Longmans, Green and co.

[13] Alexander RD 1987 The biology of moral systems. Piscataway, NJ: Transaction Publishers.

[14] Mailath GJ, Samuelson L 2006 Repeated games and reputations: long-run relationships. Oxford, UK: Oxford University Press.

[15] Gouldner AW 1960 The norm of reciprocity: a preliminary statement. Am. Sociol. Rev. 25, 161–178. (10.2307/2092623)

[16] Axelrod R, Hamilton WD 1981 The evolution of cooperation. Science 211, 1390–1396. (10.1126/science.7466396) 7466396

[17] Nowak MA, Roch S 2007 Upstream reciprocity and the evolution of gratitude. Proc. R. Soc. B 274, 605–610. (10.1098/rspb.2006.0125) PMC2197219 17254983

[18] Ostrom E 1998 A behavioral approach to the rational choice theory of collective action: presidential address, American Political Science Association, 1997. Am. Political Sci. Rev. 92, 1–22. (10.2307/2585925)

[19] Milinski M 2016 Reputation, a universal currency for human social interactions. Phil. Trans. R. Soc. B 371, 20150100 (10.1098/rstb.2015.0100) 26729939 PMC4760200

[20] Panchanathan K, Boyd R 2004 Indirect reciprocity can stabilize cooperation without the second-order free rider problem. Nature 432, 499–502. (10.1038/nature02978) 15565153

[21] Fehr E 2004 Human behaviour: don't lose your reputation. Nature 432, 449–450. (10.1038/432449a) 15565133

[22] Yoeli E, Hoffman M, Rand DG, Nowak MA 2013 Powering up with indirect reciprocity in a large-scale field experiment. Proc. Natl Acad. Sci. USA 110, 10 424–10 429. (10.1073/pnas.1301210110) PMC3690615 23754399

[23] Perc M, Gómez-Garde nes J, Szolnoki A, Floría LM, Moreno Y 2013 Evolutionary dynamics of group interactions on structured populations: a review. J. R. Soc. Interface 10, 20120997 (10.1098/rsif.2012.0997) 23303223 PMC3565747

[24] Alchian AA, Demsetz H 1972 Production, information costs, and economic organization. Am. Econ. Rev. 62, 777–795.

[25] Nowak MA, Sigmund K 1998 The dynamics of indirect reciprocity. J. Theor. Biol. 194, 561–574. (10.1006/jtbi.1998.0775) 9790830

[26] Wedekind C, Milinski M 2000 Cooperation through image scoring in humans. Science 288, 850–852. (10.1126/science.288.5467.850) 10797005

[27] Nowak MA, Sigmund K 1998 Evolution of indirect reciprocity by image scoring. Nature 393, 573–577. (10.1038/31225) 9634232

[28] Wedekind C, Braithwaite VA 2002 The long-term benefits of human generosity in indirect reciprocity. Curr. Biol. 12, 1012–1015. (10.1016/S0960-9822(02)00890-4) 12123575

[29] Semmann D, Krambeck HJ, Milinski M 2005 Reputation is valuable within and outside one's own social group. Behav. Ecol. Sociobiol. (Print) 57, 611–616. (10.1007/s00265-004-0885-3)

[30] Seinen I, Schram A 2006 Social status and group norms: indirect reciprocity in a repeated helping experiment. Eur. Econ. Rev. 50, 581–602. (10.1016/j.euroecorev.2004.10.005)

[31] Milinski M, Semmann D, Bakker TC, Krambeck H-J 2001 Cooperation through indirect reciprocity: image scoring or standing strategy? Proc. R. Soc. Lond. B 268, 2495–2501. (10.1098/rspb.2001.1809) PMC1088906 11747570

[32] Bolton GE, Katok E, Ockenfels A 2005 Cooperation among strangers with limited information about reputation. J. Public. Econ. 89, 1457–1468. (10.1016/j.jpubeco.2004.03.008)

[33] Berger U, Grüne A 2016 On the stability of cooperation under indirect reciprocity with first-order information. Games. Econ. Behav. 98, 19–33. (10.1016/j.geb.2016.05.003)

[34] Nax HH, Perc M, Szolnoki A, Helbing D 2015 Stability of cooperation under image scoring in group interactions. Sci. Rep. 5, 12145 (10.1038/srep12145) 26177466 PMC4502532

[35] Brandt H, Sigmund K 2005 Indirect reciprocity, image scoring, and moral hazard. Proc. Natl Acad. Sci. USA 102, 2666–2670. (10.1073/pnas.0407370102) 15695589 PMC548983

[36] Panchanathan K, Boyd R 2003 A tale of two defectors: the importance of standing for evolution of indirect reciprocity. J. Theor. Biol. 224, 115–126. (10.1016/S0022-5193(03)00154-1) 12900209

[37] Ohtsuki H, Iwasa Y 2004 How should we define goodness? Reputation dynamics in indirect reciprocity. J. Theor. Biol. 231, 107–120. (10.1016/j.jtbi.2004.06.005) 15363933

[38] Brandt H, Sigmund K 2004 The logic of reprobation: assessment and action rules for indirect reciprocation. J. Theor. Biol. 231, 475–486. (10.1016/j.jtbi.2004.06.032) 15488525

[39] Ohtsuki H, Iwasa Y 2006 The leading eight: social norms that can maintain cooperation by indirect reciprocity. J. Theor. Biol. 239, 435–444. (10.1016/j.jtbi.2005.08.008) 16174521

[40] Cuesta JA, Gracia-Lázaro C, Ferrer A, Moreno Y, Sánchez A 2015 Reputation drives cooperative behaviour and network formation in human groups. Sci. Rep. 5, 7843 (10.1038/srep07843) 25598347 PMC4297950

[41] Milinski M, Semmann D, Krambeck H-J 2002 Reputation helps solve the ‘tragedy of the commons’. Nature 415, 424–426. (10.1038/415424a) 11807552

[42] Brown LD, Cai TT, DasGupta A 2001 Interval estimation for a binomial proportion. Stat. Sci. 16, 101–117.

[43] Ledyard JO 1995 Public goods: a survey of experimental research. In (eds JH Kagel, AE Roth) The handbook of experimental economics, pp. 111–194. Princeton, NJ: Princeton University Press.

[44] Muller L, Sefton M, Steinberg R, Vesterlund L 2008 Strategic behavior and learning in repeated voluntary contribution experiments. J. Econ. Behav. Organ. 67, 782–793. (10.1016/j.jebo.2007.09.001)

[45] Burton-Chellew MN, Nax HH, West SA 2015 Payoff-based learning explains the decline in cooperation in public goods games. Proc. R. Soc. Lond. B 282, 20142678 (10.1098/rspb.2014.2678) PMC4309006 25589609

[46] Nax HH, Burton-Chellew MN, West SA, Young HP 2016 Learning in a black box. J. Econ. Behav. Organ. 127, 1–15. (10.1016/j.jebo.2016.04.006)

[47] Fischbacher U, Gächter S, Fehr E 2001 Are people conditionally cooperative? Evidence from a public goods experiment. Econ. Lett. 71, 397–404. (10.1016/S0165-1765(01)00394-9)

[48] Grujić J, Eke B, Cabrales A, Cuesta JA, Sánchez A 2012 Three is a crowd in iterated prisoner's dilemmas: experimental evidence on reciprocal behavior. Sci. Rep. 2, 638 (10.1038/srep00638) 22962633 PMC3435562

[49] Barcelo H, Capraro V 2015 Group size effect on cooperation in one-shot social dilemmas. Sci. Rep. 5, 7937 (10.1038/srep07937) 25605124 PMC4300455

[50] Nosenzo D, Quercia S, Sefton M 2015 Cooperation in small groups: the effect of group size. Exp. Econ. 18, 4–14. (10.1007/s10683-013-9382-8)

[51] Fischbacher U 2007 z-tree: Zurich toolbox for ready-made economic experiments. Exp. Econ. 10, 171–178. (10.1007/s10683-006-9159-4)

[52] Bock O, Baetge I, Nicklisch A 2014 hroot: Hamburg registration and organization online tool. Eur. Econ. Rev. 71, 117–120. (10.1016/j.euroecorev.2014.07.003)

[53] Mann HB, Whitney DR 1947 On a test of whether one of two random variables is stochastically larger than the other. Ann. Math. Stat. 18, 50–60. (10.1214/aoms/1177730491)

[54] Richardson AM 2015 Nonparametric statistics: a step-by-step approach. Int. Stat. Rev. 83, 163–164. (10.1111/insr.12095_3)

