# Supplementary material for: Groups and scores: the decline of cooperation
Source: J R Soc Interface. 2018 Jul 4;15(144):20180158. doi: 10.1098/rsif.2018.0158 (PMC6073651; doi:10.1098/rsif.2018.0158)
Supplement: SM [file rsif20180158supp1.pdf]

Supplementary Material:

"Groups and scores: the decline of cooperation"

Stefano Duca[1,2] and Heinrich H. Nax[1]

[1]ETH Zürich, D-GESS, 8092 Zürich , Switzerland
[2]sduca@ethz.ch

June 6, 2018

Contents

S1 Detailed experimental results **S2**

S2 Percentage of cooperators as a function of the observed score in their group **S8**

S3 Further statistical analysis **S15**

S4 Decision making micro model **S19**
S4.1 Fit for first 12 rounds S23
S4.2 Fit for all rounds S30
S4.3 Marginal effects S36
S4.4 Order Effects S39

S5 Laboratory instructions **S40**

S6 Screenshots **S41**

S7 Dataset **S50**

S8 Bibliography **S51**

S1 Detailed experimental results

In the following we show the percentage of cooperators as a function of time for each treatment and phase. The plots show data separately for the three phases: the Initial phase (denoted in green) and the first (red) and second (blue) scoring phases. We immediately observe from the pictures that there seem to be no qualitative difference between the behavior of players in the first and second scoring phase.

The errors bars indicate the 95% Binomial proportion confidence interval[1]. The difference in the error bars' size depends on the wide difference in the number of available data points[2].

Because not all treatments were played in every phase (see Table 2 in the main manuscript), not all the plots show data from all the three phases of the experiment; e.g. only the No scoring treatment was played during the initial phase and so for all the other treatments there is no plot for the Initial Phase of the experiment.

[1] For an exact definition see e.g. [1].

[2] Because we did not run experiments for all possible treatments combinations, the number of data points available for each treatment differs considerably. E.g the Image self scoring treatment was only run before the self scoring treatment, resulting in many less data points for it compared to other treatments. Furthermore, the No scoring treatment has been run at least once in every session (during the first phase), thus resulting in more available data points than all the other treatments.

No scoring

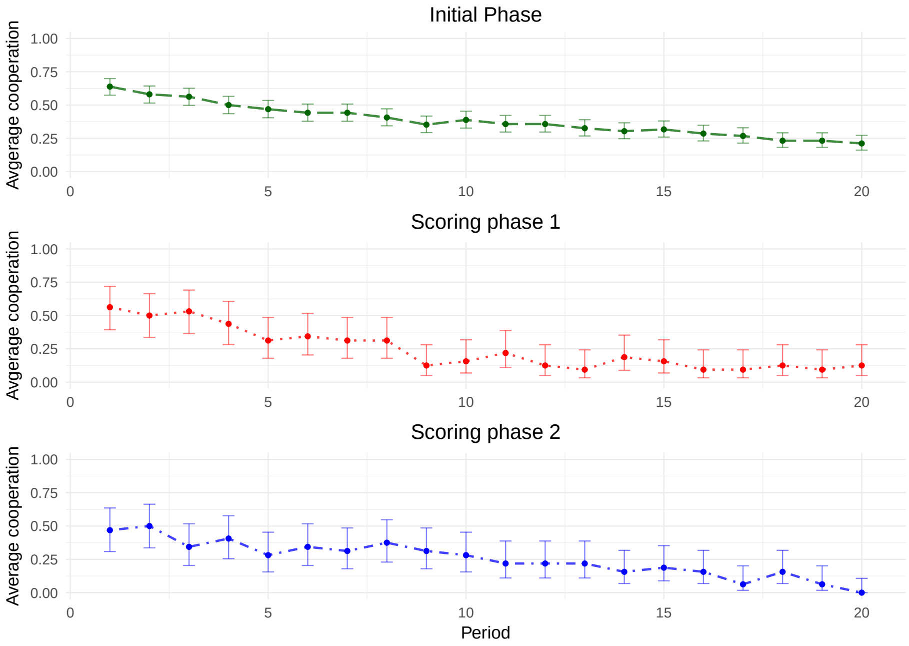

Figure S1: Average percentage of cooperators as a function of time for the No scoring treatment. The three phases are shown separately: the initial phase in green, first scoring phase in red and the second one in blue.

Image scoring

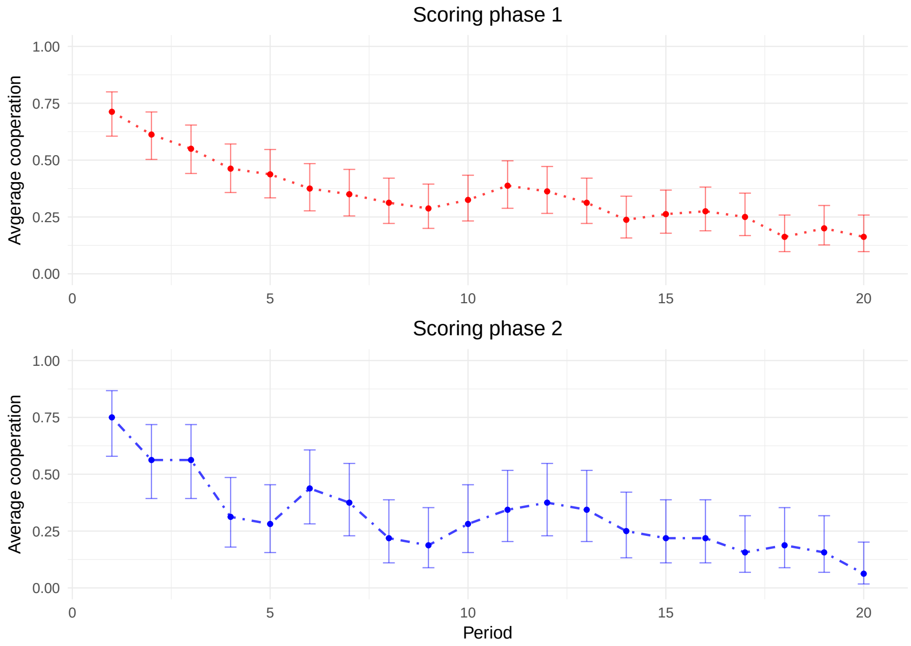

Figure S2: Average percentage of cooperators as a function of time for the Image scoring treatment. The phases are shown separately: the first scoring phase in red and the second one in blue. In no session, the Image scoring treatment was never played during the Initial phase.

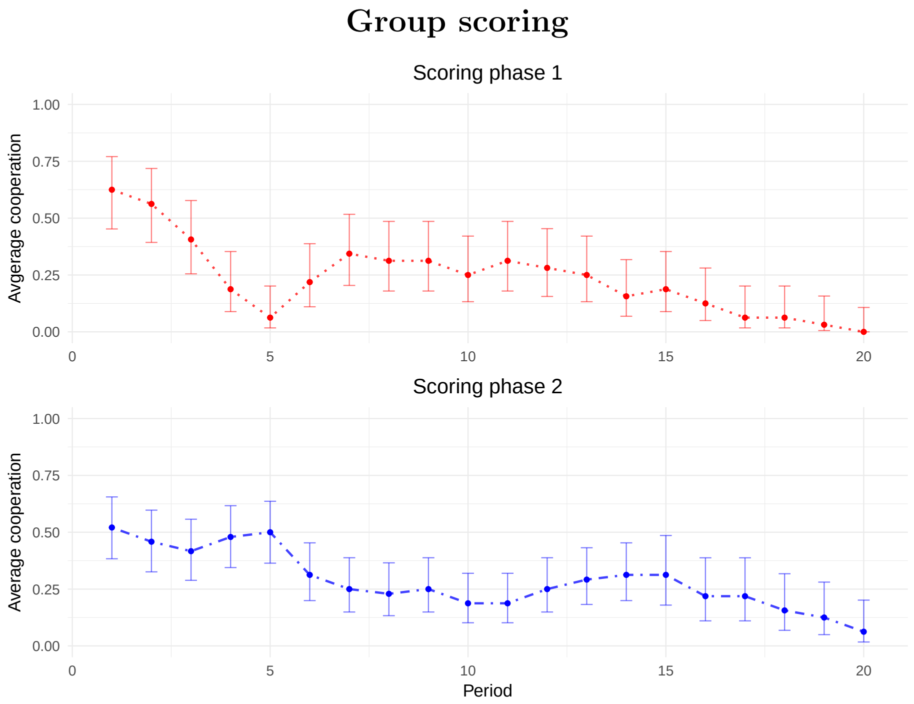

Figure S3: Average percentage of cooperators as a function of time for the Group scoring treatment. The phases are shown separately: the first scoring phase in red and the second one in blue. In no session, the Group scoring treatment was never played during the Initial phase.

Self scoring

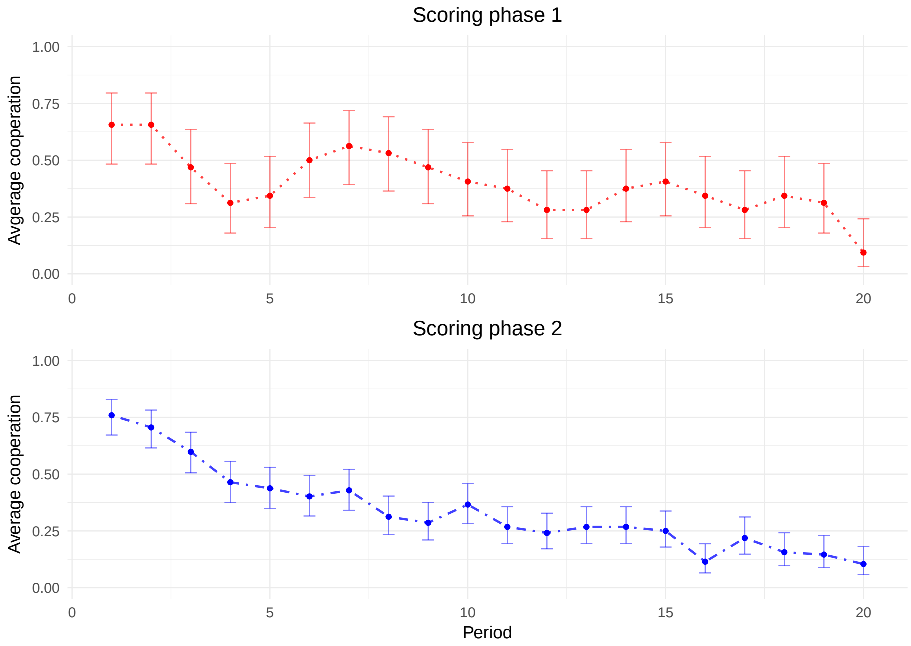

Figure S4: Average percentage of cooperators as a function of time for the Self scoring treatment. The phases are shown separately: the first scoring phase in red and the second one in blue. In no session, the Image scoring treatment was never played during the Self phase.

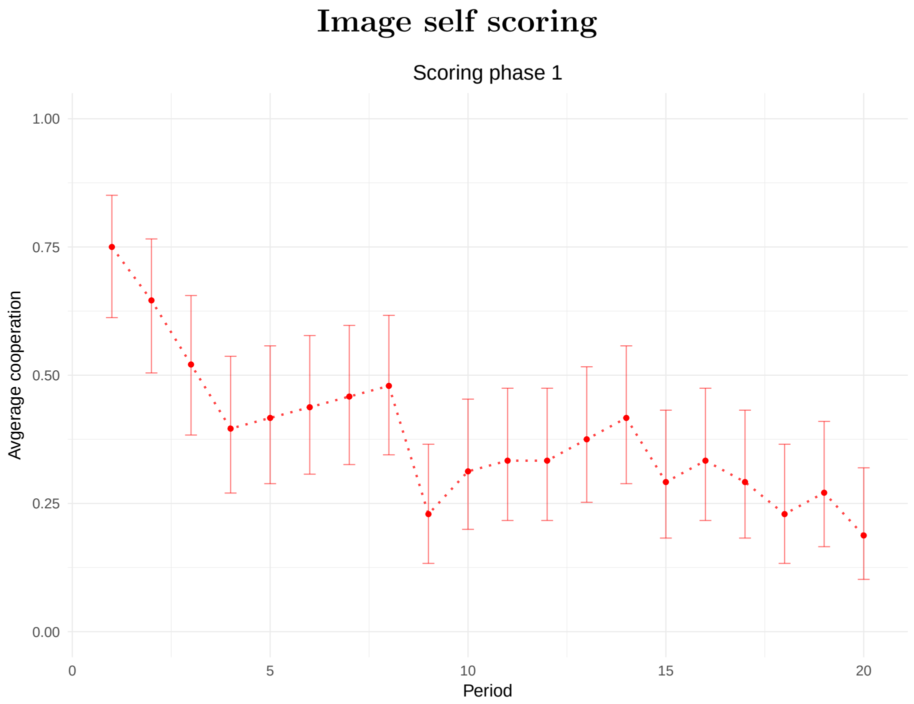

Figure S5: Average percentage of cooperators as a function of time for the Image self scoring treatment. The Image self scoring treatment was only played during the first scoring phase.

S2 Percentage of cooperators as a function of the observed score in their group

In the following, we plot the percentage of cooperators as a function of the observed score in their group.

In the Image and Group score figures, we can observe that players contribute more with increasing observed score in their group. However, players seem to do so with a downward bias, especially for high score values; this downward bias results in a steady reduction of "good players"[3] in the population and thus in the breakdown of whatever positive effect the scoring mechanism might have had on the cooperative behavior of players.

In the plots below, the bars indicate the 95% Binomial proportion confidence interval, computed using the Wilson score interval[1]. The Wilson score was chosen because it is well behaved even for small sample sizes and extreme probabilities, and it returns asymmetric confidence intervals[4].

The numbers at the side of each point indicate how many experimental points contributed to the average. The different number of observations derives from the fact that different scoring mechanisms produce a different number of possible combinations of scores in a group.

[3] I.e. players with a high score.

[4] A focal property of the Wilson score is that it returns non-vanishing error bars for small sample sizes even when the expected probability is 0 or 1.

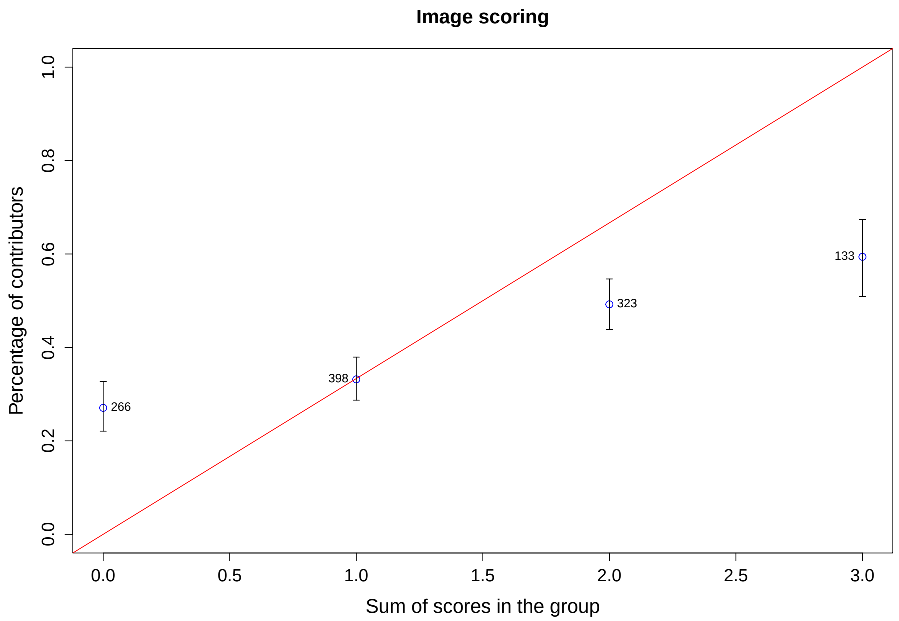

Figure S6: Cooperation as a function of observed score for the Image scoring treatment. The red line in the figure represents the identity line.

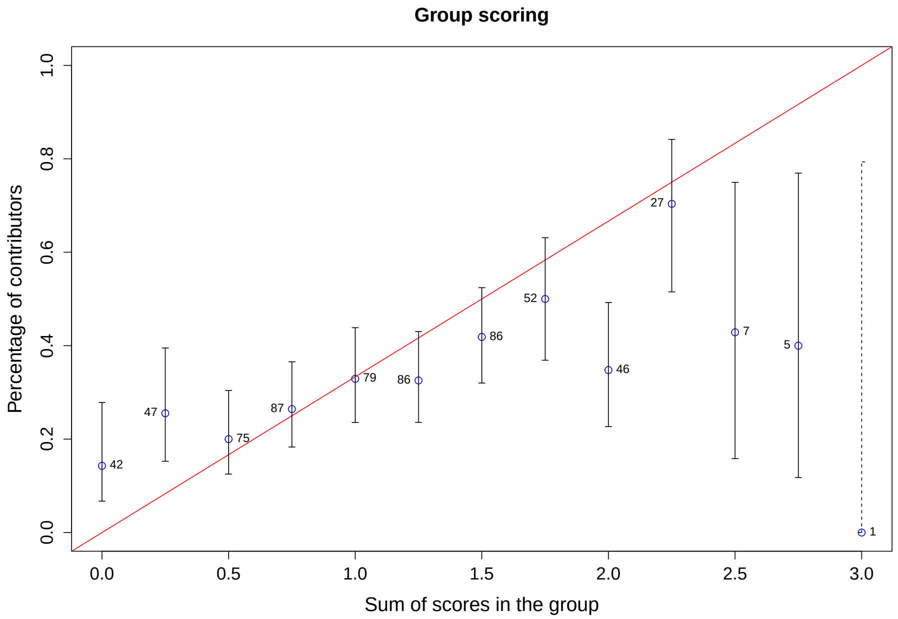

Figure S7: Cooperation as a function of observed score for the Group scoring treatment. The red line in the figure represents the identity line. The dashed error bar indicates that, due to the low number of observations for that point, it is not possible to provide a good estimation of the confidence interval; nevertheless, the best estimation is provided.

Due to the high numbers of possible values for the Self and Image Self scores, the related figures are more chaotic but a similar trend seems to be present as well.

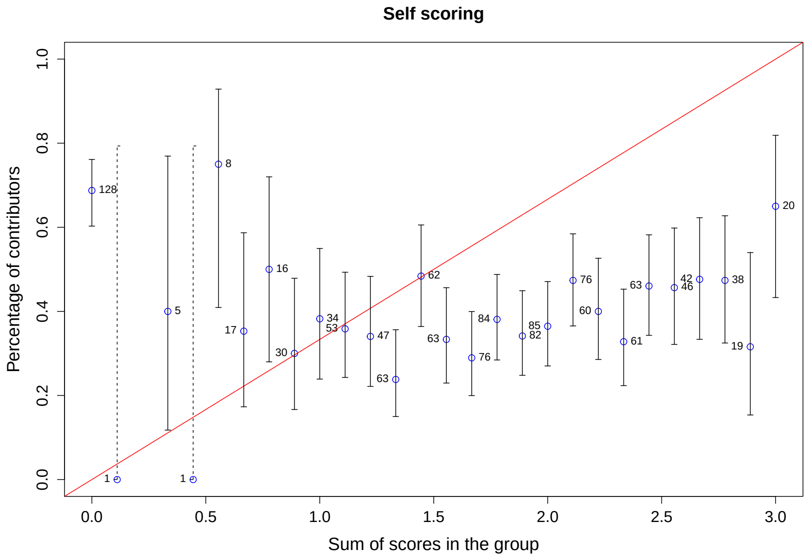

Figure S8: Cooperation as a function of observed score for the Self scoring treatment. The red line in the figure represents the identity line. The dashed error bars indicate that, due to the low number of observations for that points, it is not possible to provide a good estimation of the confidence intervals; nevertheless, the best estimations are provided.

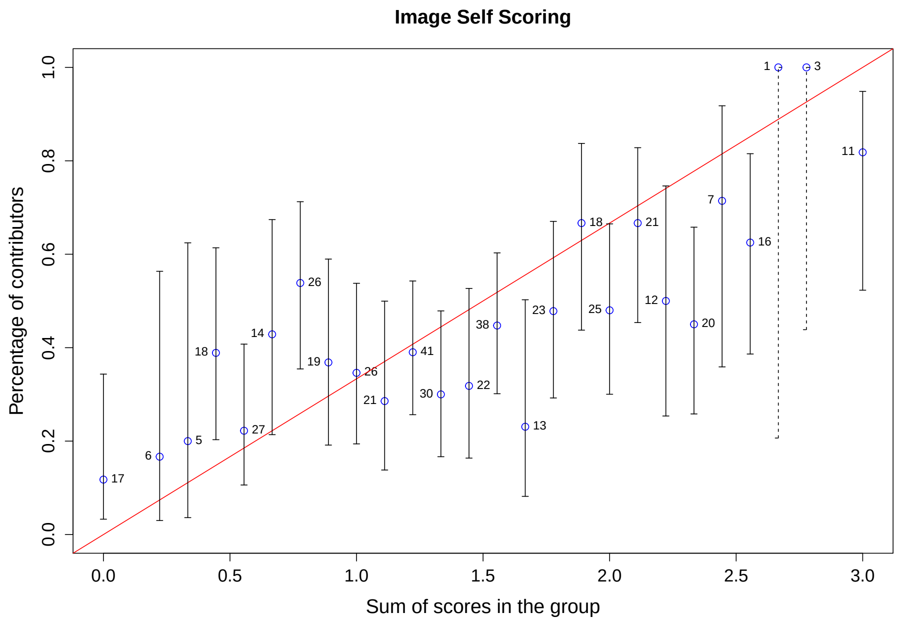

Figure S9: Cooperation as a function of observed score for the Image self scoring treatment. The red line in the figure represents the identity line. The dashed error bars indicate that, due to the low number of observations for that points, it is not possible to provide a good estimation of the confidence intervals; nevertheless, the best estimations are provided.

We now look at the case where no score at all was provided to the players: in the following we plot the percentage of cooperation in the group as a function of the image score that the players *would* have observed *if* the information would have been provided, i.e. as a function of people in the group that contributed in the previous round. In this case we observe no apparent trend of increasing cooperation.

This suggests that players were indeed responding to the observed scores in the group, and that higher levels of cooperation were not just an artifact due to the correlation between the high scores and high levels of cooperation, i.e. purely a result of conditional cooperation.

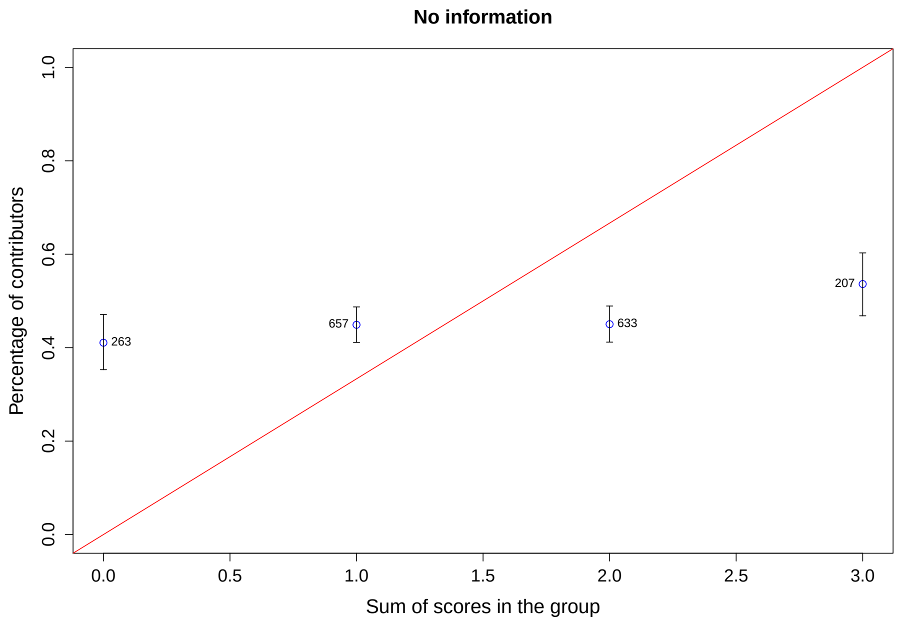

Figure S10: Cooperation as a function of what *would have been* the observed score for the No scoring treatment. The red line in the figure represents the identity line.

In order to further highlight the difference between the cases where scoring is provided or not, we also plot together the image scoring (in black) and no scoring case (in black).

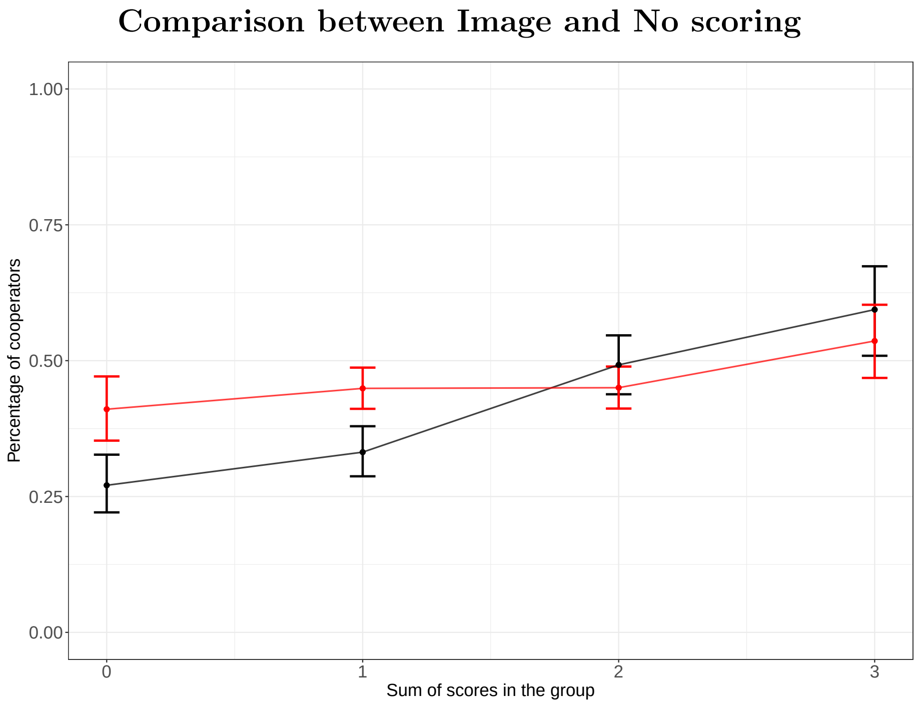

Figure S11: Comparison between the No scoring (black) and the Image scoring (red) treatments: The above picture shows the average cooperation as a function of the scores in the group. We can discern an upward trend for the Image scoring case (red line) but there seem to be no significant trend in the case of No scoring (black line). This suggests that players are indeed responding to other players' scores.

S3 Further statistical analysis

In the following, we present a different statistical analysis to test whether it is possible to statistically distinguish between treatments.

In accordance to the test presented in the main manuscript, because of the potential correlations between decisions taken by player i at time t and all decisions taken at $t-1$ (also known as temporal autocorrelations), we decided to perform the statistical test only on the data acquired during the first round of each phase. For completeness, at the end of this section we present the same test performed on the entire dataset.

Our goal is to try to understand whether the null hypothesis H_0 that treatments have no significant impact on the decisions taken by the agents is verified. For this reason, we performed a permutation test[5] using the between sum of squares (SSB) as statistics:

$$SSB = \sum_{i=1}^{m} n_i \left(\bar{y} - \bar{y}_i\right)^2$$

where i represents a treatment, $\bar{y}_i$ the average cooperation in that treatment and $\bar{y}$ the overall average cooperation.

The idea behind the test is to randomly permute treatments within sessions many times, thus creating a random sample of all the possible matches between our observable and the treatment under which they have been observed (see figure for a two dimensional example with 3 treatments tested 3 times each).

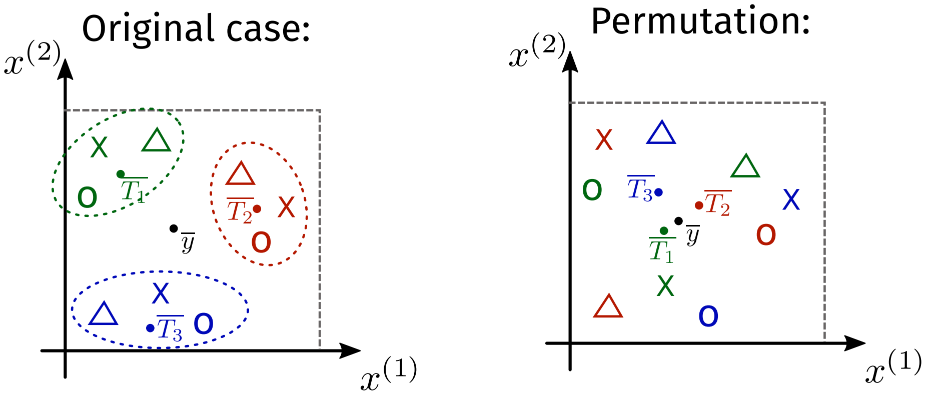

Figure S12: Example of permutation. Every symbol represents data collected in an experiment session. X, △ and O indicate that the data were collected during the first, second and third session respectively. Green indicates that data were collected during treatment 1, red during treatment 2 and blue during treatment 3. $\overline{T_i}$ is the mean for treatment i while $\bar{y}$ represents the global mean.

[5]See e.g. [2, 3, 4, 5] for a review and example applications of permutation tests.

For every random permutation we computed the resulting SSB, thus obtaining an histogram representing the empirical distribution of the SSB values.

We can now compare two hypothesis: The null hypothesis (H_0), where treatments have no impact on the decisions taken by the agents and the alternative hypothesis (H_1) where we should observe statistical significance of treatments.

To compare the two hypothesis, we calculate the SBB for our original data (let us call it SBB^*) and we compute how likely it is (using the empirical observed cumulative distribution function) to observe SBB^* under H_0.

Here is what we obtain:

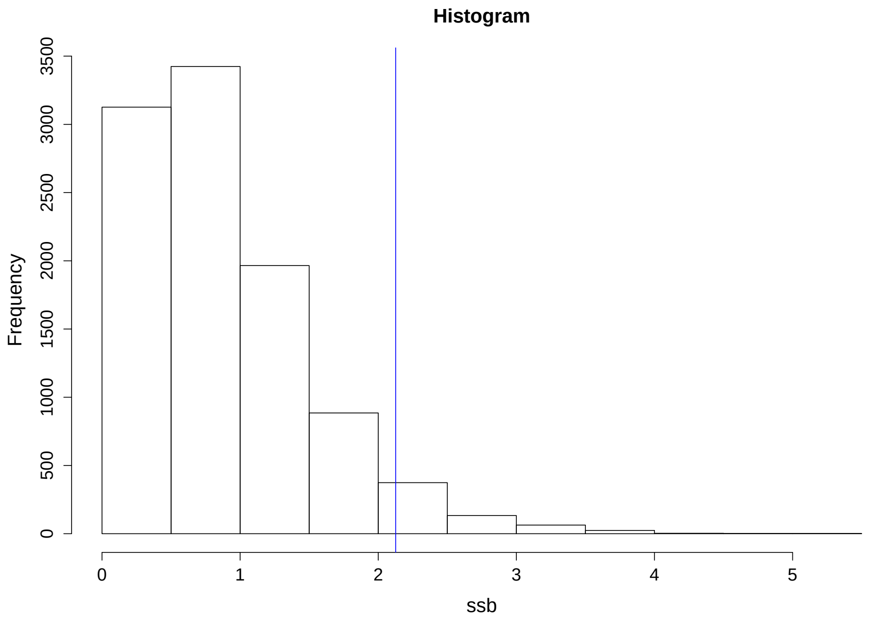

Figure S13: The histogram represents the frequency of observed sbb values obtained from 1000000 permutations. The blue line indicates where the SBB for the original data lies.

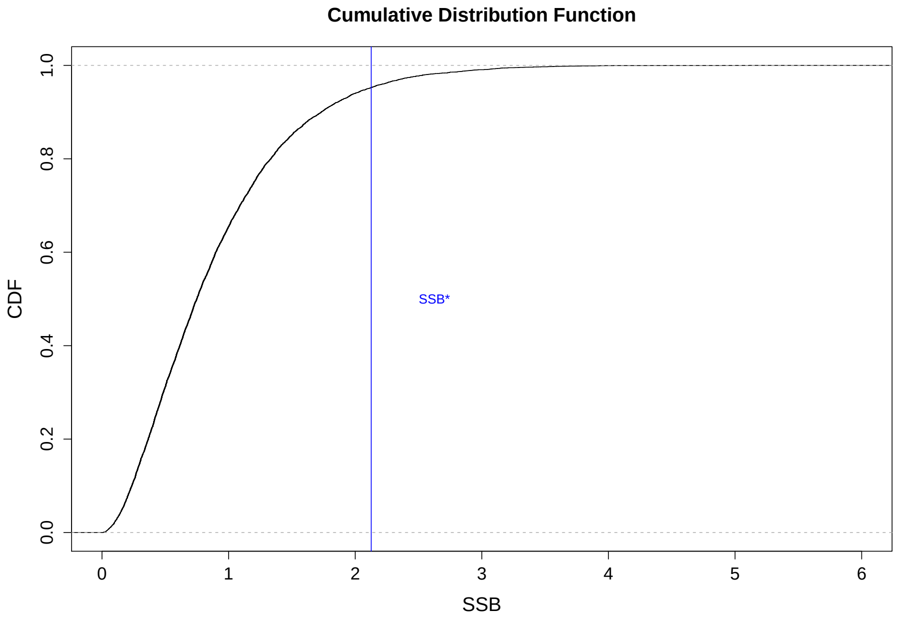

Figure S14: Here we plot the empirical cumulative distribution function obtained from the permutation. From it, it is possible to compute the empirical p-value for SBB^*.

From the figures above we can clearly observe that H_0 is rejected and thus that the treatments are not all statistically indistinguishable from each other (the observed p-value is < 0.05). This is in line with the results presented in table 3 where we observe that some treatments are significantly different from each other.

For completeness, in the following we show the histogram and empirical CDF for the entire dataset (note that some of the points in the complete dataset may be autocorrelated):

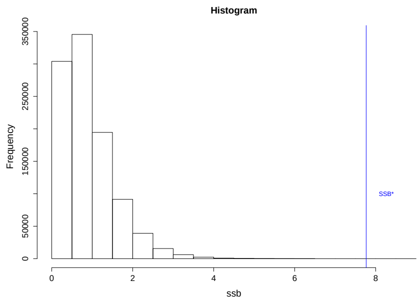

(a)

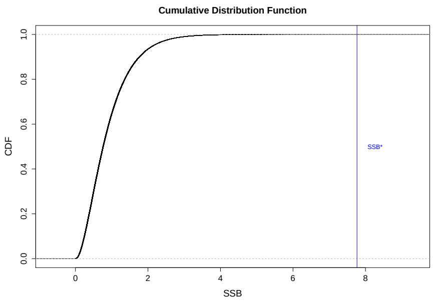

(b)

Figure S15: Histogram (a) and empirical CDF (b) obtained using the complete dataset.

S4 Decision making micro model

In the following we provide a simple model for each player's decision making and we subsequently fit it to our data.

We hypothesize that a player could act according to one or more of these behavioral rules:

- **Unconditional defection (cooperation):** Players could just unconditionally defect or cooperate, regardless of the other players' actions. We find that slightly less of 20% of the subjects in our dataset are unconditional defectors and that two players are unconditional cooperators.
- **Conditional cooperation:** Players' experiences in their previous group(s) might influence their propensity to cooperate in the future. This implies that players are sensitive to the decisions taken by other players in the past so that the more players cooperated with them in the past, the more will they tend to cooperate in the future[6]. Due to the binary nature of actions in the multi-player PD, we get non linear thresholds policies for conditional cooperation; i.e. a player might decide to cooperate if 1,2 or 3 players (other than himself) cooperated in his previous group. Figure S16 shows how a conditional cooperator would behave depending on the number of people N that cooperated in his previous group and the conditional cooperation threshold.
- **Indirect reciprocity:** Players have a propensity to cooperate with people that they know cooperated with others in the previous interaction. This means that players are sensitive to the score of the players whit which they interact. Again, the binary nature of actions results in more than threshold also for indirect reciprocity; i.e. players might decide to cooperate if the aggregated score that they observe in the group is equal or higher than different values (fixed in our case to 1,2 or 3). Figure S16 shows how an indirect reciprocator would behave depending on the observed sum of scores and his indirect reciprocity threshold.
- **Learning:** While playing, players might figure out which actions lead to higher payoff. Hence a player would decide to keep his current strategy if the payoff received in the current round is at least equal to the one received in the previous round and switch action otherwise. Figure S17 shows how a player behaving according to our definition of learning would choose his following action based on his actions in the current and previous rounds (respectively a_t and a_{t-1}) and the resulting payoffs (respectively ϕ_t and ϕ_{t-1}).

We fitted these behavioral rules to the data collected in our experiment and the results are in line with the macroscopic analysis presented in the main manuscript: We find that people significantly react to the information provided

[6]See e.g. [6, 7, 8] for theoretical and empirical results on conditional cooperation.

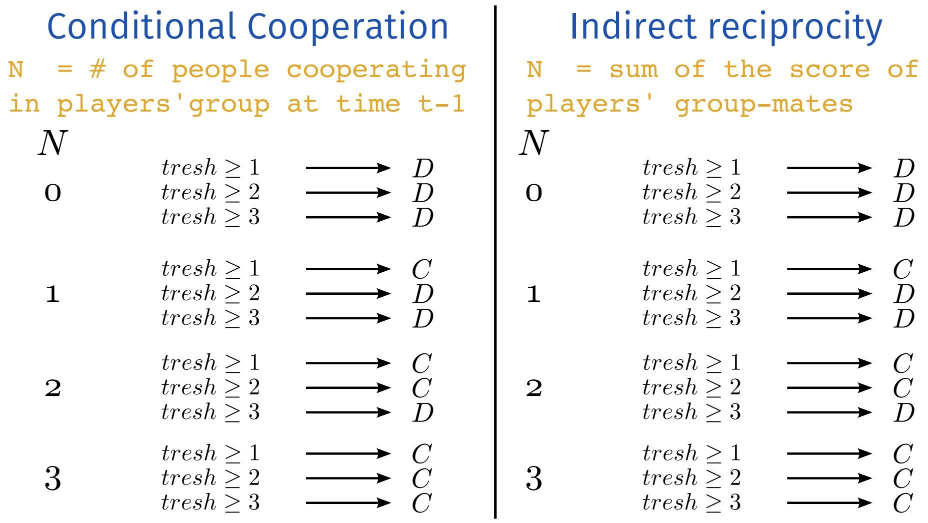

Figure S16: Predicted action for conditional cooperation (left) and indirect reciprocity (right). Due to the binary nature of actions in the multi-player PD, we obtain non linear thresholds policies for both conditional cooperation and indirect reciprocity. The decision to cooperate or not for a conditional cooperator will depend on the number of people N that cooperated in his previous group and on his conditional cooperation threshold. The decision to cooperate or not for an indirect reciprocator depends on the sum of the scores observed in his current group N and on his indirect reciprocity threshold (here set at 1,2 or 3).

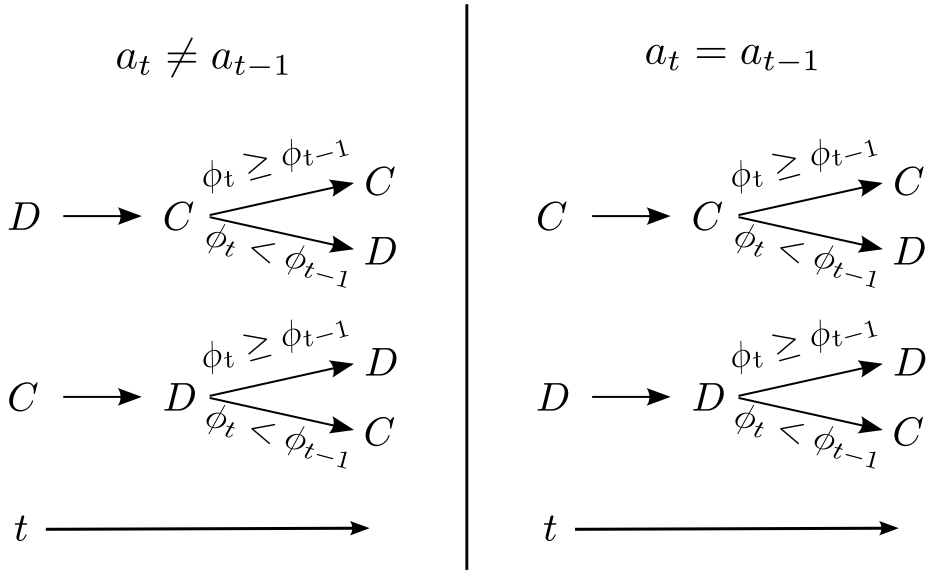

Figure S17: Predicted action for a player behaving according to (our definition) of learning. A player would decide to keep his current action ($a_t \in \{C, D\}$) if the payoff received in the current round ϕ_t is at least equal to the one received in the previous round ϕ_{t-1} and switch action otherwise.

	Learning	Cond. Coop.	Indirect Rec.
No information	✓✓	✓	NA
Image scoring	✓✓	✗	✓✓
Group scoring	✓	✗	✓
Self scoring	✓✓	✓✓	✗
Image self scoring	✓	✗	✓

Table S1: Summary of the outcome of the regressions: In the above table we summarize how significant for each treatment are Conditional Cooperation, Indirect Reciprocity and Learning. A double tick indicates that a behavioral rule is highly significant in a given treatment, a single tick signals that a rule is somehow significant and a cross indicates no evidence of that behavior in a given treatment. The results shown in the table are in agreement with the macro analysis presented in the main text: people seem to behave more according to indirect reciprocity rules, the more robust is the scoring mechanism. When scores are unreliable or not available, players revert to conditional cooperation. We also find for all treatments a significant percentage of learning behavior.

by the score. In fact, people seem to behave more according to indirect reciprocity rules, the more robust (or trustworthy) is the scoring mechanism; e.g. in the image scoring treatments players seem to rely more on an indirect reciprocity strategy than in the group scoring treatments and even more so for the self scoring treatment. When information on the score of players is either unreliable (as in the self scoring treatment) or unavailable (as in the no scoring case), people revert a conditional cooperation behavior.

Crucially, we find that players seem to have high thresholds for both conditional cooperation and indirect reciprocity. This implies that players react to other players cooperating, but not as much as they should to keep a steady percentage of cooperative players in the population. This is in perfect accord with what observed in Figure 3 in the main manuscript and it results in a steady shrinking of players with a positive score, thus negating the positive effect of reputation on cooperative behavior culminating in the ”spiraling down” of cooperation.

We also find that there is a significant percentage of learning behavior across all treatments. Table S1 summarizes our findings.

For a more detailed explanation of the regressions that we performed and for detailed results, we refer the readers to the following subsections: in subsection S4.1 we present the detailed results of regressions using the first 12 out of 20 periods and in subsection S4.2 the results using all periods. Finally, in subsection S4.3 we show the marginal effect of each independent variable over the dependent variable.

S4.1 Fit for first 12 rounds

Due to the large decline in cooperation happening during the late rounds of every treatment, it is harder to distinguish between different behavioral rules since all of the rules we consider would predict the same outcome when nearly everyone defects. For this reason, we performed our regressions using only the first 12 rounds of each phase, thus resulting in 10 data points for each phase[7]. Regressions taking into account the full dataset, are presented in the next subsection.

For every treatment, we performed a linear and a probit regression of the full model and of subsets of it.

The full model with which we fit our data is:

$$\begin{aligned} Action = \alpha + \beta_1 \cdot OneCC + \beta_2 \cdot TwoCC + \beta_3 \cdot ThreeCC + \beta_4 \cdot OneIR \\ + \beta_5 \cdot TwoIR + \beta_6 \cdot ThreeIR + \beta_7 \cdot Learn\_Inert + \beta_8 \cdot tindex \end{aligned} \quad (1)$$

where

- **Action** is the dependent variable. It can only take two values: either defect or cooperate.
- **OneCC** is a dummy variable signaling whether at least one person cooperated in the group of the active player in the previous round.
- **TwoCC** is a dummy variable signaling whether at least two people cooperated in the group of the active player in the previous round.
- **ThreeCC** is a dummy variable signaling whether at least three people cooperated in the group of the active player in the previous round.
- **OneIR** is a dummy variable signaling whether the sum of the scores in the active player's group (excluding the active player's score) is at least one.
- **TwoIR** is a dummy variable signaling whether the sum of the scores in the active player's group (excluding the active player's score) is at least two.
- **ThreeIR** is a dummy variable signaling whether the sum of the scores in the active player's group (excluding the active player's score) is at least three.
- **LearnInert** is a dummy variable taking signaling whether or not the player's choice is consistent with the learning behavior (as defined above).
- **tindex** represents the round in which the decision takes place. It ranges from 3 to 12.

[7] The predictions of the behavioral rules can be tested starting from round 3, because one needs a_t and a_{t-1} to predict the action at a_{t+1}.

Below are the results of the regressions that we performed, presented using the *texreg*[9] package. For every treatment we performed a linear and a probit regression for the entire model and then a probit regression for subsets of the model, i.e. taking into account only conditional cooperation or indirect reciprocity (OnlyCC & OnlyIR) or only one of the three possible thresholds (Only1, Only2, Only3).

No scoring

	Probit	Linear	OnlyCC	Only1	Only2	Only3
(Intercept)	-0.12	0.45^{***}	-0.12	-0.09	0.04	0.10
	(0.09)	(0.03)	(0.09)	(0.08)	(0.07)	(0.06)
OneCC	0.18^{**}	0.06^{**}	0.18^{**}	0.22^{***}		
	(0.06)	(0.02)	(0.06)	(0.06)		
TwoCC	0.08	0.03	0.08		0.16^{***}	
	(0.05)	(0.02)	(0.05)		(0.05)	
ThreeCC	0.22^{*}	0.08^{*}	0.22^{*}			0.30^{***}
	(0.09)	(0.03)	(0.09)			(0.09)
Learn_Inert	0.37^{***}	0.14^{***}	0.37^{***}	0.38^{***}	0.37^{***}	0.37^{***}
	(0.04)	(0.02)	(0.04)	(0.04)	(0.04)	(0.04)
tindex	-0.06^{***}	-0.02^{***}	-0.06^{***}	-0.07^{***}	-0.07^{***}	-0.07^{***}
	(0.01)	(0.00)	(0.01)	(0.01)	(0.01)	(0.01)
AIC	4786.39		4786.39	4793.83	4797.24	4798.37
BIC	4823.73		4823.73	4818.72	4822.13	4823.26
Log Likelihood	-2387.19		-2387.19	-2392.91	-2394.62	-2395.18
Deviance	4774.39		4774.39	4785.83	4789.24	4790.37
Num. obs.	3726	3726	3726	3726	3726	3726
R^2		0.06				
Adj. R^2		0.06				
RMSE		0.47				

$^{***}p < 0.001$, $^{**}p < 0.01$, $^{*}p < 0.05$

Table S2: Regression results for the No scoring treatment when only the first 12 rounds are taken into account. Due to the lack of information about the scores of players in this treatment, the model considered here doesn't contain the indirect reciprocity dummy variables.

Image scoring

	Probit	Linear	OnlyCC	OnlyIR	Only1	Only2	Only3
(Intercept)	-0.76^{***}	0.22^{***}	-0.12	-0.94^{***}	-0.56^{**}	-0.78^{***}	-0.50^{***}
	(0.18)	(0.06)	(0.15)	(0.16)	(0.17)	(0.15)	(0.13)
OneCC	-0.23^{*}	-0.07^{*}	-0.12		-0.10		
	(0.10)	(0.04)	(0.10)		(0.09)		
TwoCC	-0.02	-0.00	0.12			-0.04	
	(0.11)	(0.04)	(0.10)			(0.09)	
ThreeCC	0.08	0.02	0.27				0.11
	(0.16)	(0.05)	(0.15)				(0.14)
OneIR	0.14	0.04		0.12	0.60^{***}		
	(0.13)	(0.04)		(0.12)	(0.11)		
TwoIR	0.65^{***}	0.23^{***}		0.63^{***}		0.82^{***}	
	(0.11)	(0.04)		(0.10)		(0.09)	
ThreeIR	0.42^{***}	0.16^{***}		0.40^{***}			0.76^{***}
	(0.12)	(0.04)		(0.12)			(0.11)
Learn_Inert	0.42^{***}	0.14^{***}	0.45^{***}	0.44^{***}	0.45^{***}	0.45^{***}	0.44^{***}
	(0.08)	(0.03)	(0.08)	(0.08)	(0.08)	(0.08)	(0.08)
tindex	-0.01	-0.00	-0.04^{**}	-0.00	-0.04^{**}	-0.01	-0.02
	(0.02)	(0.01)	(0.01)	(0.02)	(0.01)	(0.01)	(0.01)
AIC	1275.68		1376.72	1275.54	1350.92	1285.87	1326.07
BIC	1320.38		1406.52	1305.34	1375.75	1310.70	1350.90
Log Likelihood	-628.84		-682.36	-631.77	-670.46	-637.93	-658.03
Deviance	1257.68		1364.72	1263.54	1340.92	1275.87	1316.07
Num. obs.	1060	1060	1060	1060	1060	1060	1060
R^2		0.15					
Adj. R^2		0.14					
RMSE		0.45					

$^{***}p < 0.001$, $^{**}p < 0.01$, $^{*}p < 0.05$

Table S3: Regression results for the Image scoring treatment when only the first 12 rounds are taken into account.

Group scoring

	Probit	Linear	OnlyCC	OnlyIR	Only1	Only2	Only3
(Intercept)	-0.81^{***}	0.21^{***}	-0.54^{**}	-0.79^{***}	-0.77^{***}	-0.55^{***}	-0.30^{*}
	(0.19)	(0.06)	(0.17)	(0.18)	(0.19)	(0.16)	(0.14)
OneCC	0.09	0.03	0.23^{*}		0.13		
	(0.12)	(0.04)	(0.11)		(0.11)		
TwoCC	−0.07	−0.02	0.12			−0.02	
	(0.14)	(0.05)	(0.13)			(0.12)	
ThreeCC	−0.23	−0.09	−0.02				−0.01
	(0.25)	(0.09)	(0.24)				(0.23)
OneIR	0.26^{*}	0.08^{*}		0.28^{*}	0.38^{***}		
	(0.12)	(0.04)		(0.11)	(0.11)		
TwoIR	0.49^{***}	0.19^{***}		0.46^{***}		0.61^{***}	
	(0.14)	(0.05)		(0.13)		(0.13)	
ThreeIR	0.51	0.20		0.42			0.82^{*}
	(0.40)	(0.14)		(0.39)			(0.39)
Learn_Inert	0.26^{*}	0.09^{*}	0.30^{**}	0.25^{*}	0.29^{**}	0.25^{*}	0.27^{**}
	(0.10)	(0.03)	(0.10)	(0.10)	(0.10)	(0.10)	(0.10)
tindex	−0.02	−0.01	-0.04^{*}	−0.01	−0.02	−0.02	-0.04^{*}
	(0.02)	(0.01)	(0.02)	(0.02)	(0.02)	(0.02)	(0.02)
AIC	910.91		931.29	906.62	919.01	911.74	932.04
BIC	952.73		959.17	934.50	942.24	934.97	955.27
Log Likelihood	−446.46		−459.65	−447.31	−454.50	−450.87	−461.02
Deviance	892.91		919.29	894.62	909.01	901.74	922.04
Num. obs.	770	770	770	770	770	770	770
R^2		0.07					
Adj. R^2		0.06					
RMSE		0.45					

$^{***}p < 0.001$, $^{**}p < 0.01$, $^{*}p < 0.05$

Table S4: Regression results for the Group scoring treatment when only the first 12 rounds are taken into account.

Self-scoring

	Probit	Linear	OnlyCC	OnlyIR	Only1	Only2	Only3
(Intercept)	-0.12	0.45^{***}	-0.24	0.14	-0.07	-0.01	-0.03
	(0.24)	(0.09)	(0.14)	(0.23)	(0.24)	(0.14)	(0.12)
OneCC	0.33^{***}	0.12^{***}	0.33^{***}		0.36^{***}		
	(0.10)	(0.03)	(0.10)		(0.09)		
TwoCC	-0.08	-0.03	-0.08			0.16^{*}	
	(0.09)	(0.03)	(0.09)			(0.07)	
ThreeCC	0.40^{***}	0.15^{***}	0.41^{***}				0.43^{***}
	(0.12)	(0.04)	(0.12)				(0.11)
OneIR	-0.17	-0.06		-0.12	-0.13		
	(0.21)	(0.08)		(0.21)	(0.21)		
TwoIR	0.01	0.01		0.04		0.05	
	(0.08)	(0.03)		(0.08)		(0.08)	
ThreeIR	0.08	0.03		0.14			0.10
	(0.10)	(0.04)		(0.10)			(0.09)
Learn_Inert	0.36^{***}	0.13^{***}	0.36^{***}	0.37^{***}	0.39^{***}	0.35^{***}	0.33^{***}
	(0.07)	(0.03)	(0.07)	(0.07)	(0.07)	(0.07)	(0.07)
tindex	-0.05^{***}	-0.02^{***}	-0.05^{***}	-0.06^{***}	-0.06^{***}	-0.06^{***}	-0.05^{***}
	(0.01)	(0.01)	(0.01)	(0.01)	(0.01)	(0.01)	(0.01)
AIC	1766.59		1761.84	1788.08	1771.58	1783.82	1771.26
BIC	1813.53		1793.13	1819.37	1797.66	1809.90	1797.34
Log Likelihood	-874.30		-874.92	-888.04	-880.79	-886.91	-880.63
Deviance	1748.59		1749.84	1776.08	1761.58	1773.82	1761.26
Num. obs.	1360	1360	1360	1360	1360	1360	1360
R^2		0.07					
Adj. R^2		0.06					
RMSE		0.48					

$^{***}p < 0.001$, $^{**}p < 0.01$, $^{*}p < 0.05$

Table S5: Regression results for the Self scoring treatment when only the first 12 rounds are taken into account.

Image self-scoring

	Probit	Linear	OnlyCC	OnlyIR	Only1	Only2	Only3
(Intercept)	-0.48	0.32^{***}	-0.18	-0.54^{*}	-0.32	-0.53^{*}	-0.15
	(0.26)	(0.09)	(0.23)	(0.25)	(0.25)	(0.22)	(0.18)
OneCC	-0.22	-0.08	-0.08		-0.01		
	(0.18)	(0.06)	(0.16)		(0.16)		
TwoCC	0.23	0.08	0.31^{*}			0.22	
	(0.16)	(0.06)	(0.15)			(0.13)	
ThreeCC	0.15	0.05	0.31				0.38
	(0.22)	(0.08)	(0.21)				(0.20)
OneIR	0.12	0.04		0.10	0.32		
	(0.18)	(0.06)		(0.17)	(0.17)		
TwoIR	0.45^{**}	0.17^{**}		0.49^{**}		0.50^{***}	
	(0.15)	(0.06)		(0.15)		(0.14)	
ThreeIR	0.31	0.11		0.38			0.52
	(0.29)	(0.10)		(0.28)			(0.28)
Learn_Inert	0.33^{*}	0.12^{*}	0.37^{**}	0.37^{**}	0.44^{***}	0.37^{**}	0.37^{**}
	(0.13)	(0.05)	(0.13)	(0.13)	(0.13)	(0.13)	(0.13)
tindex	-0.01	-0.00	-0.04	-0.01	-0.04	-0.01	-0.04
	(0.02)	(0.01)	(0.02)	(0.02)	(0.02)	(0.02)	(0.02)
AIC	585.61		593.47	583.71	597.94	581.26	592.25
BIC	622.59		618.13	608.37	618.49	601.80	612.80
Log Likelihood	-283.80		-290.74	-285.86	-293.97	-285.63	-291.13
Deviance	567.61		581.47	571.71	587.94	571.26	582.25
Num. obs.	450	450	450	450	450	450	450
R^2		0.10					
Adj. R^2		0.08					
RMSE		0.47					

$^{***}p < 0.001$, $^{**}p < 0.01$, $^{*}p < 0.05$

Table S6: Regression results for the Image self scoring treatment when only the first 12 rounds are taken into account.

S4.2 Fit for all rounds

In this sections we present the results of the regressions when the entire dataset is taken into consideration. The model with which we fit the data is exactly the same as the one presented in the previous subsection (except, of course, for *tindex* that in this case ranges from 3 to 20).

No scoring

	Probit	Linear	OnlyCC	Only1	Only2	Only3
(Intercept)	-0.24	0.41^{***}	-0.24	-0.19	0.03	0.02
	(0.14)	(0.05)	(0.14)	(0.13)	(0.12)	(0.11)
OneCC	0.33^{***}	0.12^{***}	0.33^{***}	0.35^{***}		
	(0.10)	(0.03)	(0.10)	(0.09)		
TwoCC	-0.08	-0.03	-0.08		0.16^{*}	
	(0.09)	(0.03)	(0.09)		(0.07)	
ThreeCC	0.41^{***}	0.16^{***}	0.41^{***}			0.44^{***}
	(0.12)	(0.04)	(0.12)			(0.11)
Learn_Inert	0.36^{***}	0.13^{***}	0.36^{***}	0.39^{***}	0.35^{***}	0.33^{***}
	(0.07)	(0.03)	(0.07)	(0.07)	(0.07)	(0.07)
tindex	-0.05^{***}	-0.02^{***}	-0.05^{***}	-0.06^{***}	-0.06^{***}	-0.06^{***}
	(0.01)	(0.00)	(0.01)	(0.01)	(0.01)	(0.01)
AIC	1761.84		1761.84	1769.99	1782.18	1770.32
BIC	1793.13		1793.13	1790.85	1803.05	1791.18
Log Likelihood	-874.92		-874.92	-880.99	-887.09	-881.16
Deviance	1749.84		1749.84	1761.99	1774.18	1762.32
Num. obs.	1360	1360	1360	1360	1360	1360
R^2		0.07				
Adj. R^2		0.06				
RMSE		0.48				

$^{***}p < 0.001$, $^{**}p < 0.01$, $^{*}p < 0.05$

Table S7: Regression results for the No scoring treatment when all the rounds are taken into account. Due to the lack of information about the scores of players in this treatment, the model considered here doesn't contain the indirect reciprocity dummy variables.

Image scoring

	Probit	Linear	OnlyCC	OnlyIR	Only1	Only2	Only3
(Intercept)	-0.76^{***}	0.22^{***}	-0.12	-0.94^{***}	-0.56^{**}	-0.78^{***}	-0.50^{***}
	(0.18)	(0.06)	(0.15)	(0.16)	(0.17)	(0.15)	(0.13)
OneCC	-0.23^{*}	-0.07^{*}	-0.12		-0.10		
	(0.10)	(0.04)	(0.10)		(0.09)		
TwoCC	-0.02	-0.00	0.12			-0.04	
	(0.11)	(0.04)	(0.10)			(0.09)	
ThreeCC	0.08	0.02	0.27				0.11
	(0.16)	(0.05)	(0.15)				(0.14)
OneIR	0.14	0.04		0.12	0.60^{***}		
	(0.13)	(0.04)		(0.12)	(0.11)		
TwoIR	0.65^{***}	0.23^{***}		0.63^{***}		0.82^{***}	
	(0.11)	(0.04)		(0.10)		(0.09)	
ThreeIR	0.42^{***}	0.16^{***}		0.40^{***}			0.76^{***}
	(0.12)	(0.04)		(0.12)			(0.11)
Learn_Inert	0.42^{***}	0.14^{***}	0.45^{***}	0.44^{***}	0.45^{***}	0.45^{***}	0.44^{***}
	(0.08)	(0.03)	(0.08)	(0.08)	(0.08)	(0.08)	(0.08)
tindex	-0.01	-0.00	-0.04^{**}	-0.00	-0.04^{**}	-0.01	-0.02
	(0.02)	(0.01)	(0.01)	(0.02)	(0.01)	(0.01)	(0.01)
AIC	1275.68		1376.72	1275.54	1350.92	1285.87	1326.07
BIC	1320.38		1406.52	1305.34	1375.75	1310.70	1350.90
Log Likelihood	-628.84		-682.36	-631.77	-670.46	-637.93	-658.03
Deviance	1257.68		1364.72	1263.54	1340.92	1275.87	1316.07
Num. obs.	1060	1060	1060	1060	1060	1060	1060
R^2		0.15					
Adj. R^2		0.14					
RMSE		0.45					

$^{***}p < 0.001$, $^{**}p < 0.01$, $^{*}p < 0.05$

Table S8: Regression results for the Image scoring treatment when all the rounds are taken into account.

Group scoring

	Probit	Linear	OnlyCC	OnlyIR	Only1	Only2	Only3
(Intercept)	-0.81^{***}	0.21^{***}	-0.54^{**}	-0.79^{***}	-0.77^{***}	-0.55^{***}	-0.30^{*}
	(0.19)	(0.06)	(0.17)	(0.18)	(0.19)	(0.16)	(0.14)
OneCC	0.09	0.03	0.23^{*}		0.13		
	(0.12)	(0.04)	(0.11)		(0.11)		
TwoCC	−0.07	−0.02	0.12			−0.02	
	(0.14)	(0.05)	(0.13)			(0.12)	
ThreeCC	−0.23	−0.09	−0.02				−0.01
	(0.25)	(0.09)	(0.24)				(0.23)
OneIR	0.26^{*}	0.08^{*}		0.28^{*}	0.38^{***}		
	(0.12)	(0.04)		(0.11)	(0.11)		
TwoIR	0.49^{***}	0.19^{***}		0.46^{***}		0.61^{***}	
	(0.14)	(0.05)		(0.13)		(0.13)	
ThreeIR	0.51	0.20		0.42			0.82^{*}
	(0.40)	(0.14)		(0.39)			(0.39)
Learn_Inert	0.26^{*}	0.09^{*}	0.30^{**}	0.25^{*}	0.29^{**}	0.25^{*}	0.27^{**}
	(0.10)	(0.03)	(0.10)	(0.10)	(0.10)	(0.10)	(0.10)
tindex	−0.02	−0.01	-0.04^{*}	−0.01	−0.02	−0.02	-0.04^{*}
	(0.02)	(0.01)	(0.02)	(0.02)	(0.02)	(0.02)	(0.02)
AIC	910.91		931.29	906.62	919.01	911.74	932.04
BIC	952.73		959.17	934.50	942.24	934.97	955.27
Log Likelihood	−446.46		−459.65	−447.31	−454.50	−450.87	−461.02
Deviance	892.91		919.29	894.62	909.01	901.74	922.04
Num. obs.	770	770	770	770	770	770	770
R^2		0.07					
Adj. R^2		0.06					
RMSE		0.45					

$^{***}p < 0.001$, $^{**}p < 0.01$, $^{*}p < 0.05$

Table S9: Regression results for the Group scoring treatment when all the rounds are taken into account.

Self-scoring

	Probit	Linear	OnlyCC	OnlyIR	Only1	Only2	Only3
(Intercept)	-0.12	0.45^{***}	-0.24	0.14	-0.07	-0.01	-0.03
	(0.24)	(0.09)	(0.14)	(0.23)	(0.24)	(0.14)	(0.12)
OneCC	0.33^{***}	0.12^{***}	0.33^{***}		0.36^{***}		
	(0.10)	(0.03)	(0.10)		(0.09)		
TwoCC	-0.08	-0.03	-0.08			0.16^{*}	
	(0.09)	(0.03)	(0.09)			(0.07)	
ThreeCC	0.40^{***}	0.15^{***}	0.41^{***}				0.43^{***}
	(0.12)	(0.04)	(0.12)				(0.11)
OneIR	-0.17	-0.06		-0.12	-0.13		
	(0.21)	(0.08)		(0.21)	(0.21)		
TwoIR	0.01	0.01		0.04		0.05	
	(0.08)	(0.03)		(0.08)		(0.08)	
ThreeIR	0.08	0.03		0.14			0.10
	(0.10)	(0.04)		(0.10)			(0.09)
Learn_Inert	0.36^{***}	0.13^{***}	0.36^{***}	0.37^{***}	0.39^{***}	0.35^{***}	0.33^{***}
	(0.07)	(0.03)	(0.07)	(0.07)	(0.07)	(0.07)	(0.07)
tindex	-0.05^{***}	-0.02^{***}	-0.05^{***}	-0.06^{***}	-0.06^{***}	-0.06^{***}	-0.05^{***}
	(0.01)	(0.01)	(0.01)	(0.01)	(0.01)	(0.01)	(0.01)
AIC	1766.59		1761.84	1788.08	1771.58	1783.82	1771.26
BIC	1813.53		1793.13	1819.37	1797.66	1809.90	1797.34
Log Likelihood	-874.30		-874.92	-888.04	-880.79	-886.91	-880.63
Deviance	1748.59		1749.84	1776.08	1761.58	1773.82	1761.26
Num. obs.	1360	1360	1360	1360	1360	1360	1360
R^2		0.07					
Adj. R^2		0.06					
RMSE		0.48					

$^{***}p < 0.001$, $^{**}p < 0.01$, $^{*}p < 0.05$

Table S10: Regression results for the Self scoring treatment when all the rounds are taken into account.

Image self-scoring

	Probit	Linear	OnlyCC	OnlyIR	Only1	Only2	Only3
(Intercept)	-0.48	0.32^{***}	-0.18	-0.54^{*}	-0.32	-0.53^{*}	-0.15
	(0.26)	(0.09)	(0.23)	(0.25)	(0.25)	(0.22)	(0.18)
OneCC	-0.22	-0.08	-0.08		-0.01		
	(0.18)	(0.06)	(0.16)		(0.16)		
TwoCC	0.23	0.08	0.31^{*}			0.22	
	(0.16)	(0.06)	(0.15)			(0.13)	
ThreeCC	0.15	0.05	0.31				0.38
	(0.22)	(0.08)	(0.21)				(0.20)
OneIR	0.12	0.04		0.10	0.32		
	(0.18)	(0.06)		(0.17)	(0.17)		
TwoIR	0.45^{**}	0.17^{**}		0.49^{**}		0.50^{***}	
	(0.15)	(0.06)		(0.15)		(0.14)	
ThreeIR	0.31	0.11		0.38			0.52
	(0.29)	(0.10)		(0.28)			(0.28)
Learn_Inert	0.33^{*}	0.12^{*}	0.37^{**}	0.37^{**}	0.44^{***}	0.37^{**}	0.37^{**}
	(0.13)	(0.05)	(0.13)	(0.13)	(0.13)	(0.13)	(0.13)
tindex	-0.01	-0.00	-0.04	-0.01	-0.04	-0.01	-0.04
	(0.02)	(0.01)	(0.02)	(0.02)	(0.02)	(0.02)	(0.02)
AIC	585.61		593.47	583.71	597.94	581.26	592.25
BIC	622.59		618.13	608.37	618.49	601.80	612.80
Log Likelihood	-283.80		-290.74	-285.86	-293.97	-285.63	-291.13
Deviance	567.61		581.47	571.71	587.94	571.26	582.25
Num. obs.	450	450	450	450	450	450	450
R^2		0.10					
Adj. R^2		0.08					
RMSE		0.47					

$^{***}p < 0.001$, $^{**}p < 0.01$, $^{*}p < 0.05$

Table S11: Regression results for the Image Self scoring treatment when all the rounds are taken into account.

S4.3 Marginal effects

In the following subsection, we plot the marginal effects of each independent variable over the dependent variable, i.e. the choice to cooperate or defect in the following round, as obtained from the probit regression[8].

For each treatment, we compute the average of the sample marginal effects, as suggested in Fernihough[10].

The results are shown below; the error bars indicate the confidence interval at 2σ.

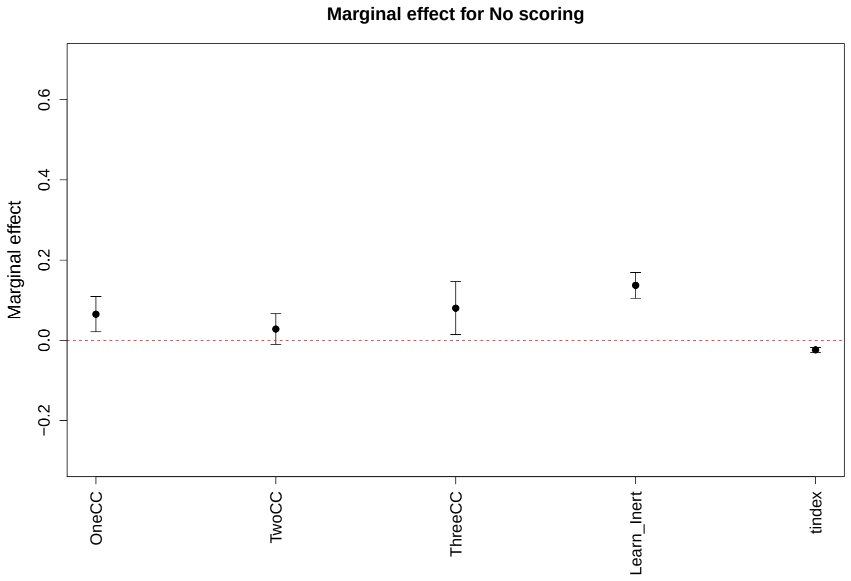

Figure S18: Marginal effects of all the independent variables for the No scoring treatment. Due to the lack of information about the scores of players in this treatment, the model considered here doesn't contain the indirect reciprocity dummy variables.

[8]The marginal effect m of an independent variable x on a dependent variable y can be interpreted as the value such that a unit increase in x increases y by m units.

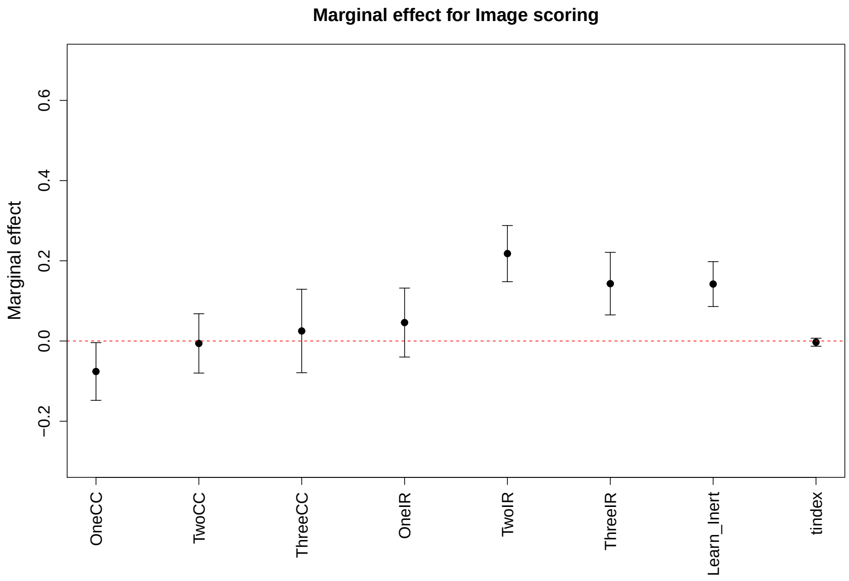

Figure S19: Marginal effects of all the independent variables for the Image scoring treatment.

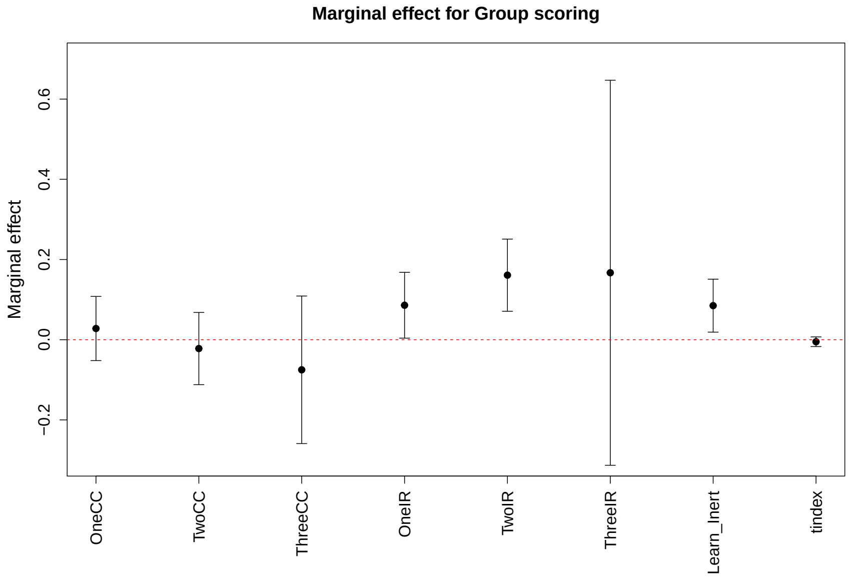

Figure S20: Marginal effects of all the independent variables for the Group scoring treatment.

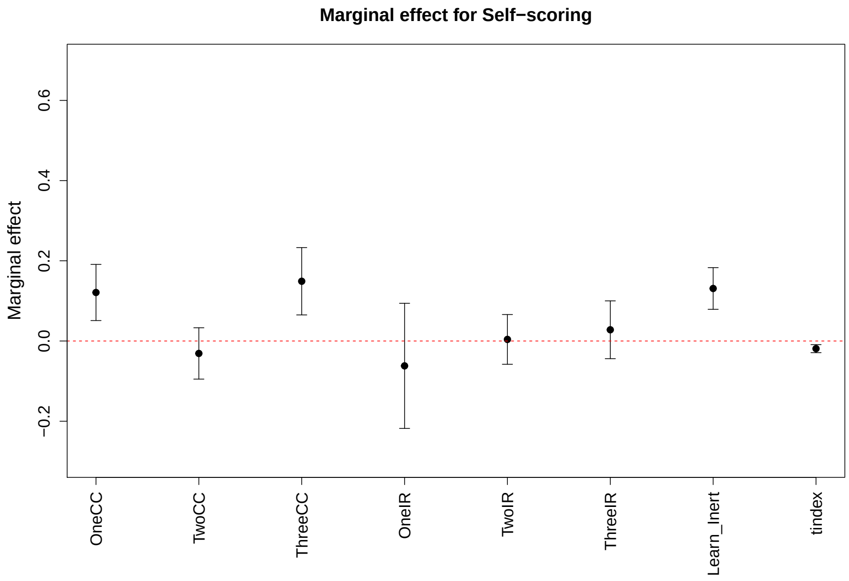

Figure S21: Marginal effects of all the independent variables for the Self scoring treatment.

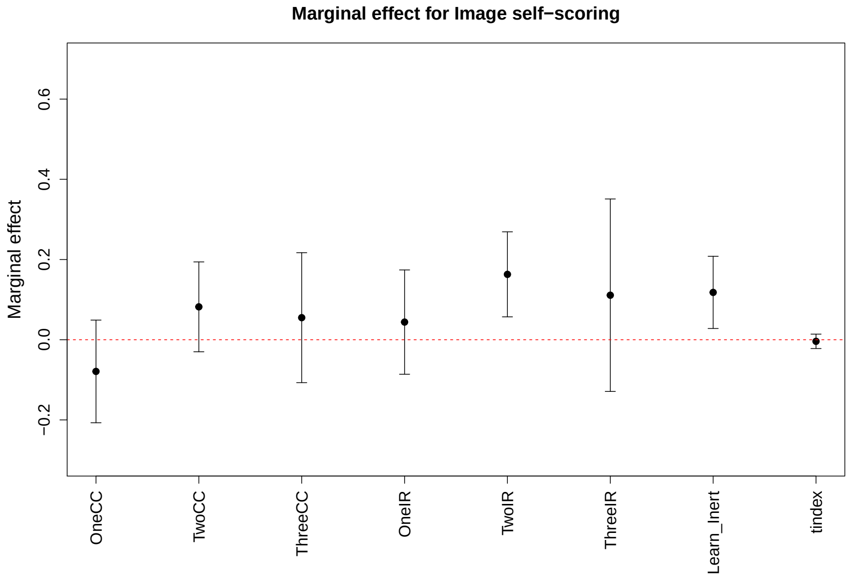

Figure S22: Marginal effects of all the independent variables for the Image self scoring treatment.

S4.4 Order Effects

Throughout both this and the main manuscript, we have assumed that the order in which treatments are played have no influence over the decision of the players. This assumption was supported by qualitative observation of our data and the well known restart effect (see e.g. Andreoni[11]).

Here we very briefly check whether this assumption holds by performing a very simple linear regression with the order of the treatment as the only independent variable and the player's decision as dependent variable:

	Order linear regression
(Intercept)	0.41^{***}
	(0.01)
Order	-0.03^{***}
	(0.01)
R^2	0.00
Adj. R^2	0.00
Num. obs.	13248
RMSE	0.47

$^{***}p < 0.001$, $^{**}p < 0.01$, $^{*}p < 0.05$

Table S12: Results of a linear regression assuming the order in which treatments are played as the only independent variable.

As we can observe from the above table, the order in which the treatments are played results to be significant. However, the marginal effect of it seems to be quite small[9].

Hence, we conclude that, while the order is not completely irrelevant, its effect on the overall decision of the players are small enough that our approximation remains reasonable.

[9]In a linear regression, the coefficient of the regression is a good approximation of the marginal effect of an independent variable.

S5 Laboratory instructions

As part of the supplementary material, we provide all the written instructions that were handed out to the players during the experiment. The instructions are provided as a separate (*tar*) file.

The instructions consisted in a welcome/introductory sheet that was given to all players when they arrived in the laboratory and in treatment-specific instructions, given to the players at the beginning of every phase. The instructions given to the players at the beginning of the phases varied depending on which treatment the players had already played during the session. Hence each set of instructions is labeled as $i-j-k$ (with $i, j, k \in \{1, \ldots, 5\}$) where i, j, k indicate which treatments were played during that session and $i-j-k$ indicates the order in which they were played.

The mapping from numbers to treatments is the following:

1. No scoring
2. Image scoring
3. Group scoring
4. Self scoring
5. Image self scoring

No further instructions, other than the written ones, were provided to the subjects.

S6 Screenshots

In the following we show screenshots of various stages of the experiment. These are exactly what the participants saw on their screens.

Payoff calculator

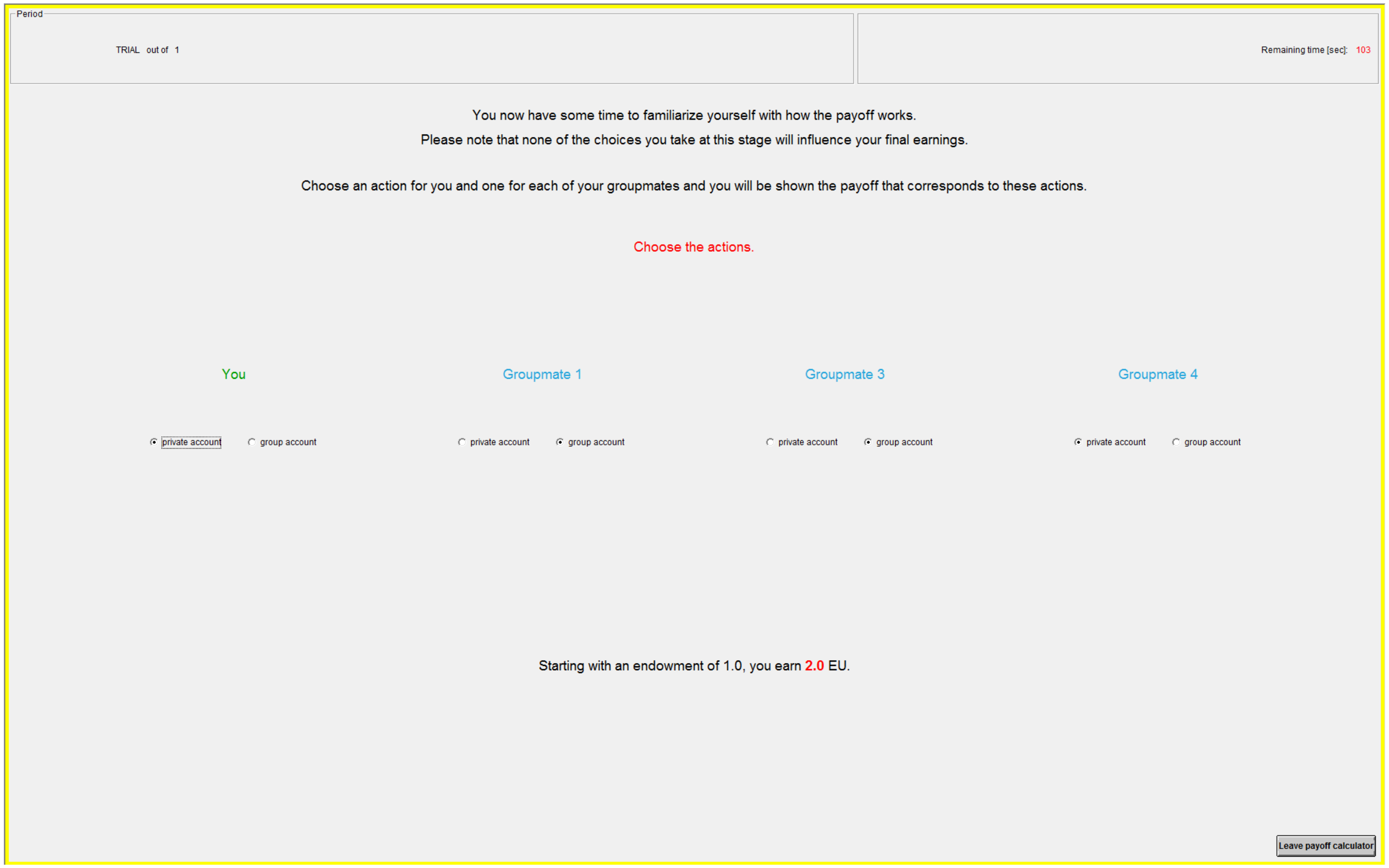

Figure S23: This is a screenshot of the payoff calculator. In every session, before the Initial Phase, players were given time to understand the game by trying different combinations of theirs and their teammates actions and observe the payoff outcome.

Figure S24: This is

Initial phase

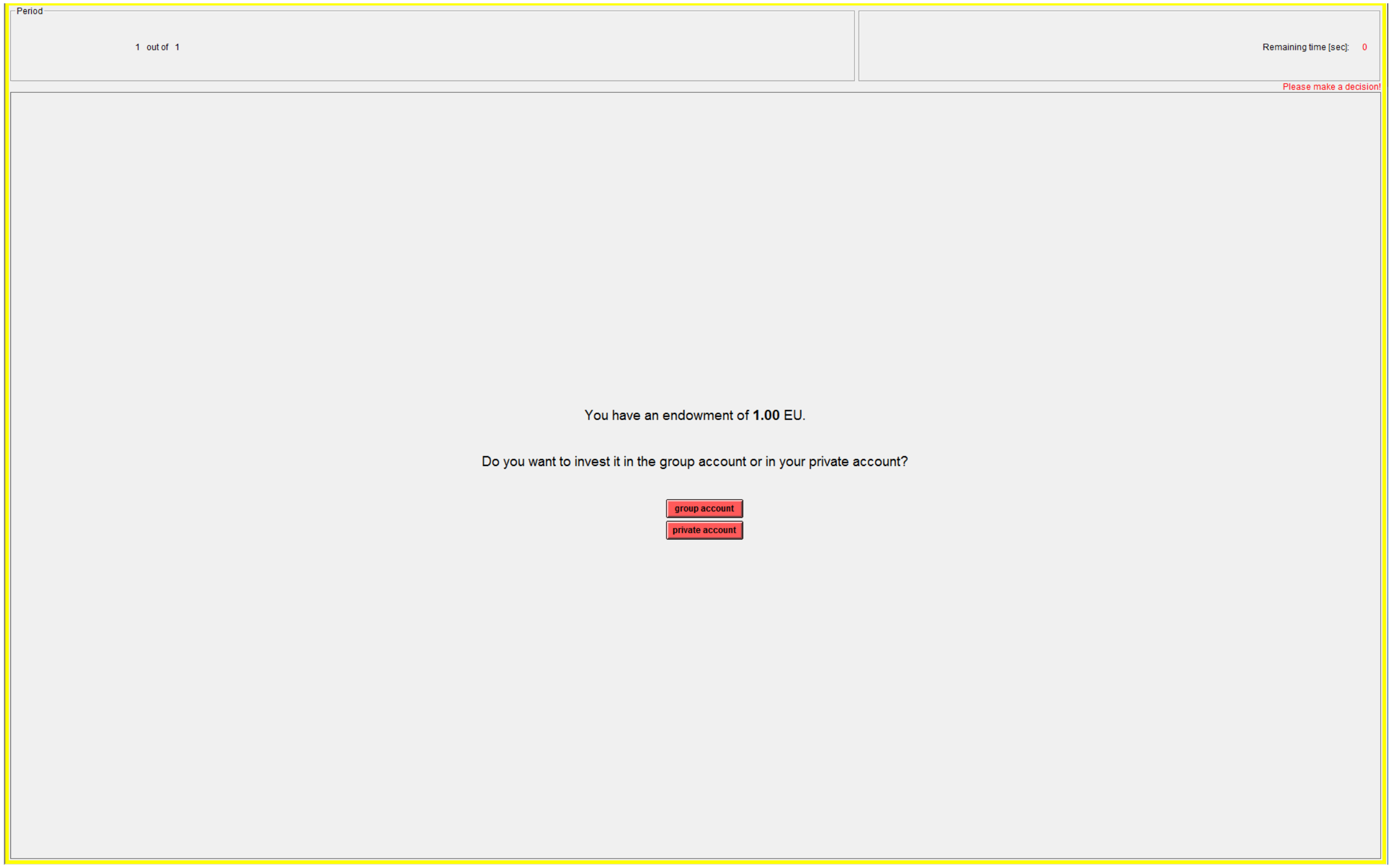

Figure S25: This is the decision screen shown to the players during the initial phase. As shown by the screenshot, no information regarding the teammates is provided to the players.

Feedback

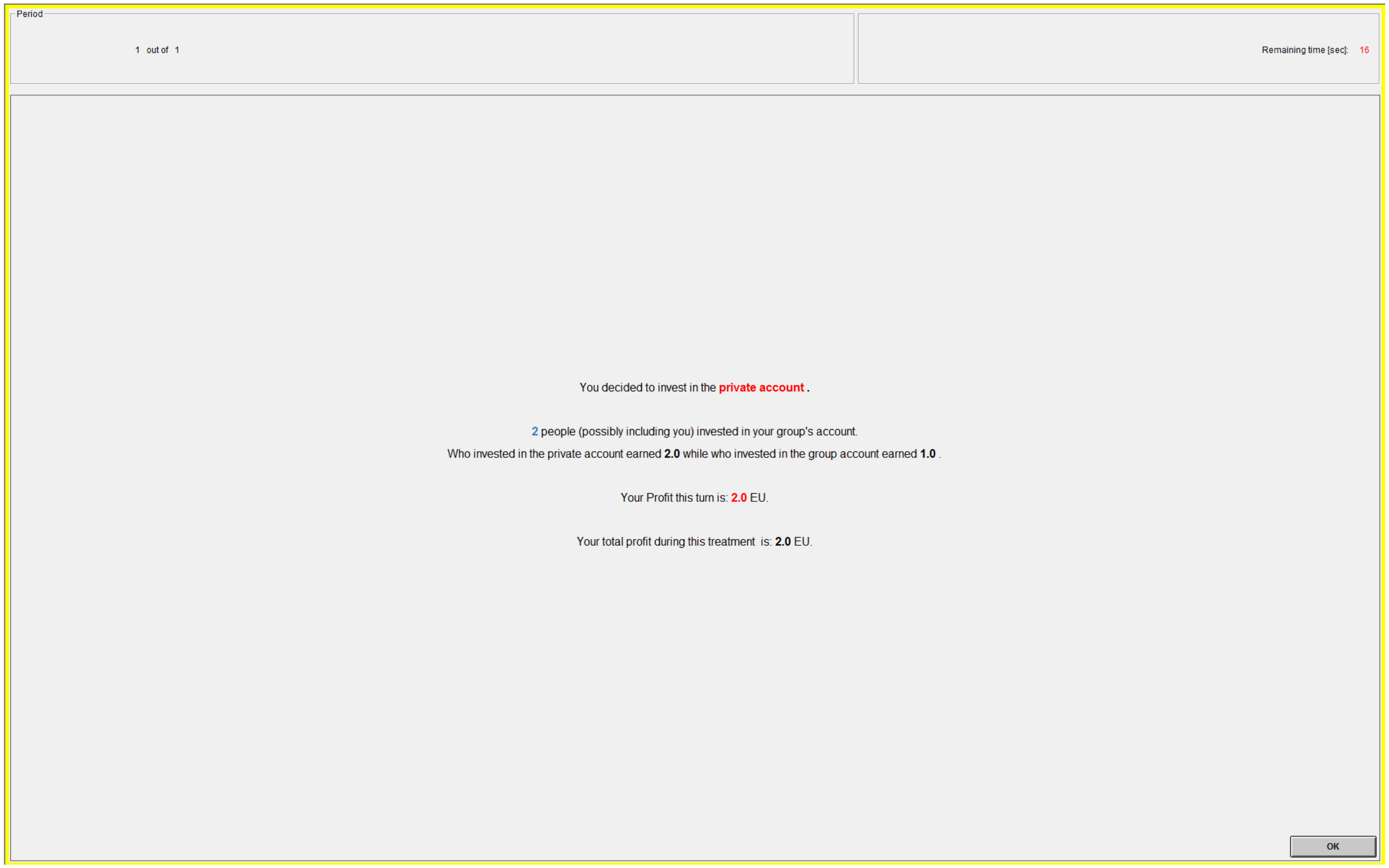

Figure S26: This is the feedback screen as shown in all treatments other than Self Scoring. It shows how many players defected or cooperated in the player's group and his/her resulting payoff for the round.

Image scoring

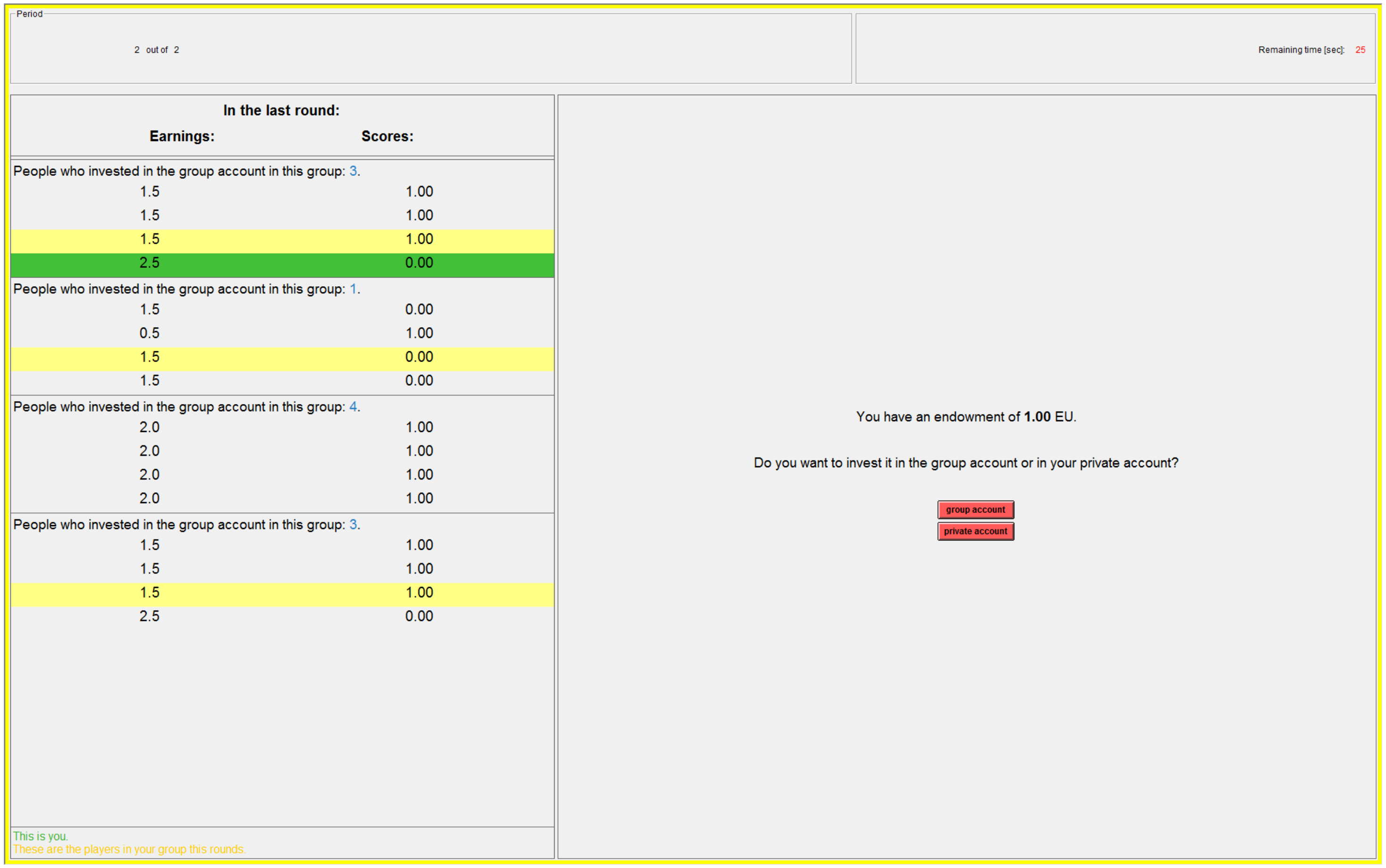

Figure S27: This is the decision screen shown to the players in the Image Scoring treatment (from the second round on). On the left it shows how players where grouped in the previous round and what where the individual decisions. The green line highlights the player and the yellow lines the player's teammates in the current round.

Group scoring

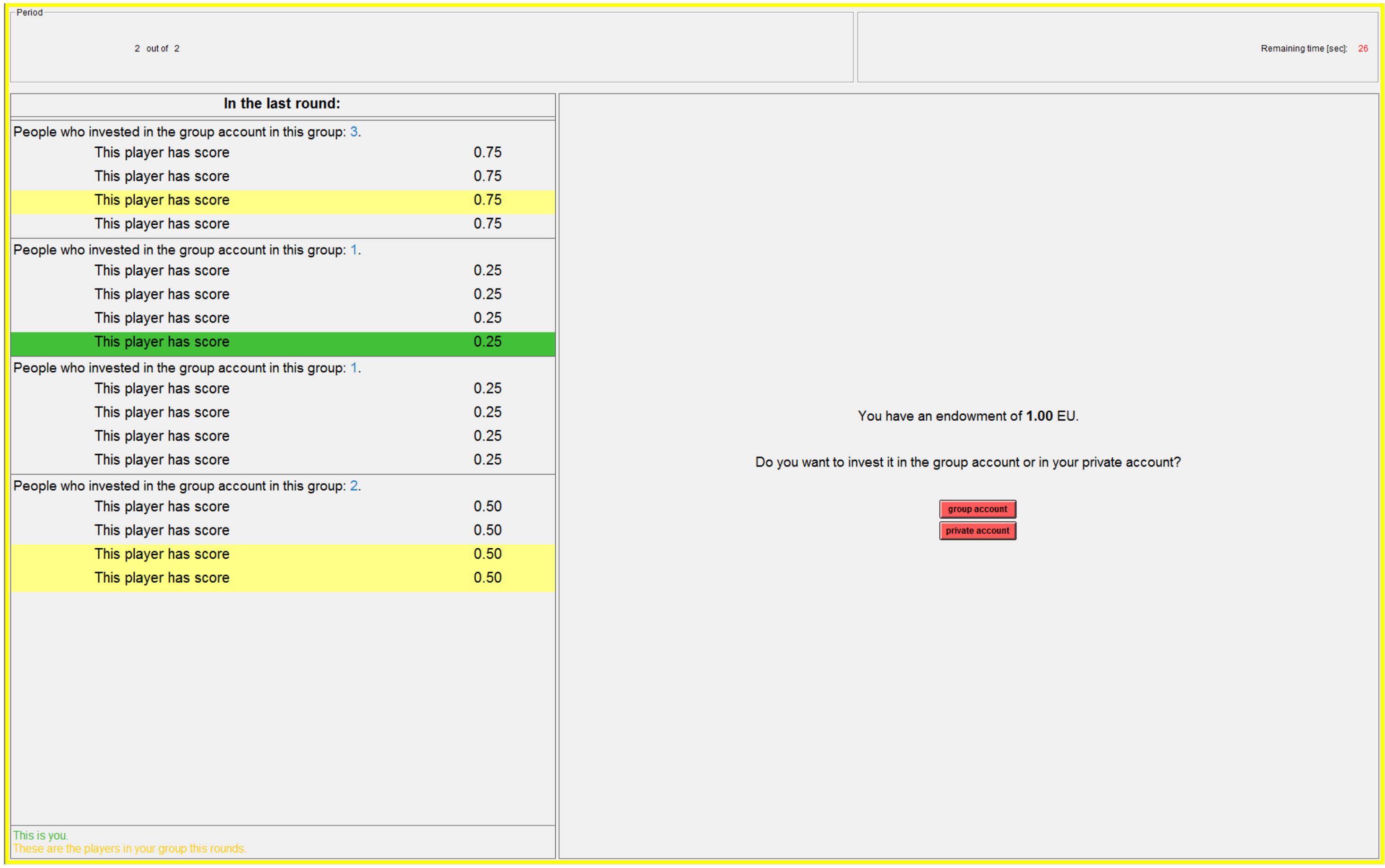

Figure S28: This is the decision screen shown to the players in the Group Scoring treatment (from the second round on). On the left it shows how players where grouped in the previous round and the group score of each group. The green line highlights the player and the yellow lines the player's teammates in the current round.

Self scoring

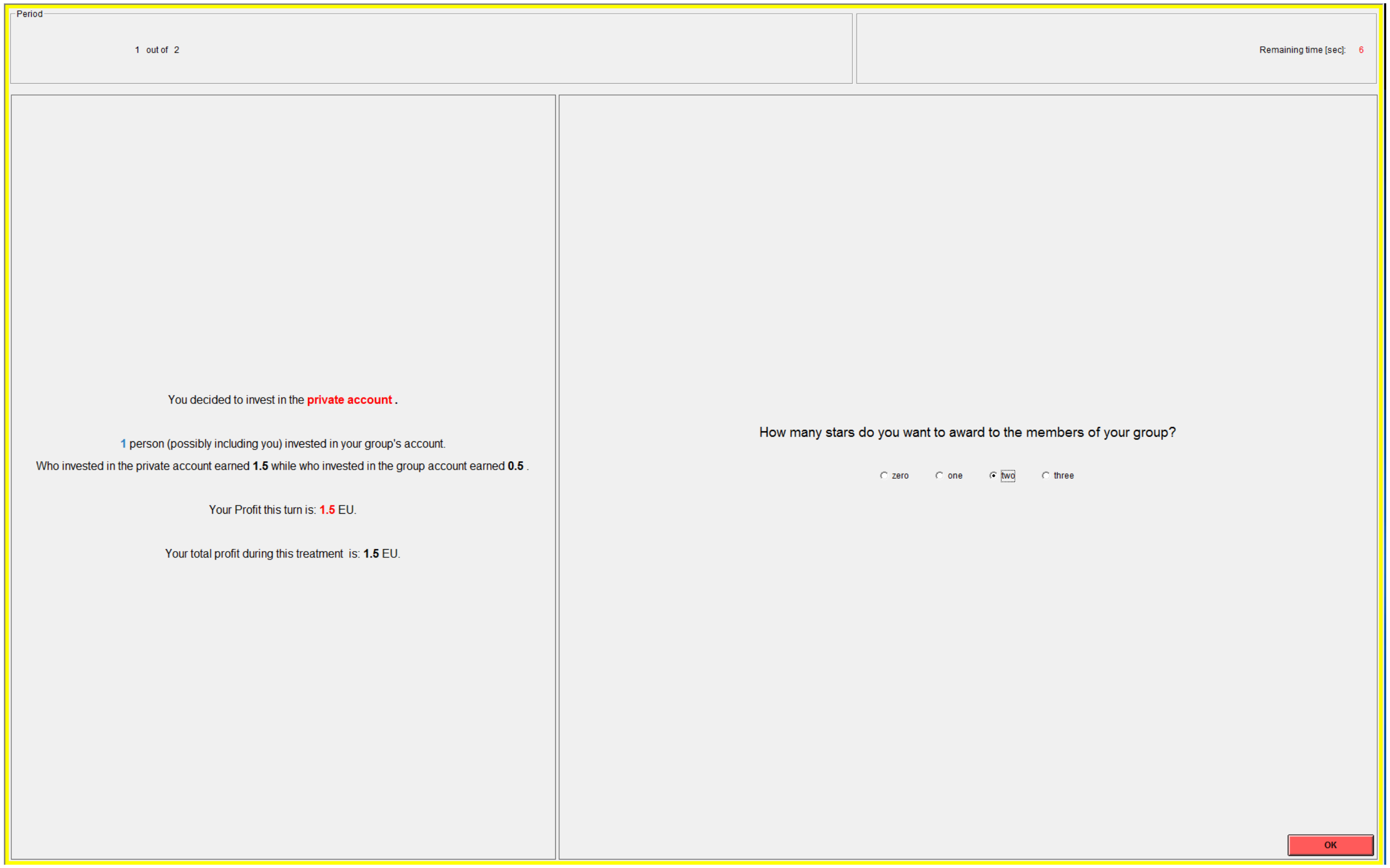

Figure S29: This is the feedback screen shown to the players in the Self Scoring treatment . In addition to receive the same feedback as in all the other treatments, players are asked to rate their group on a scale from zero to three.

Image Self scoring

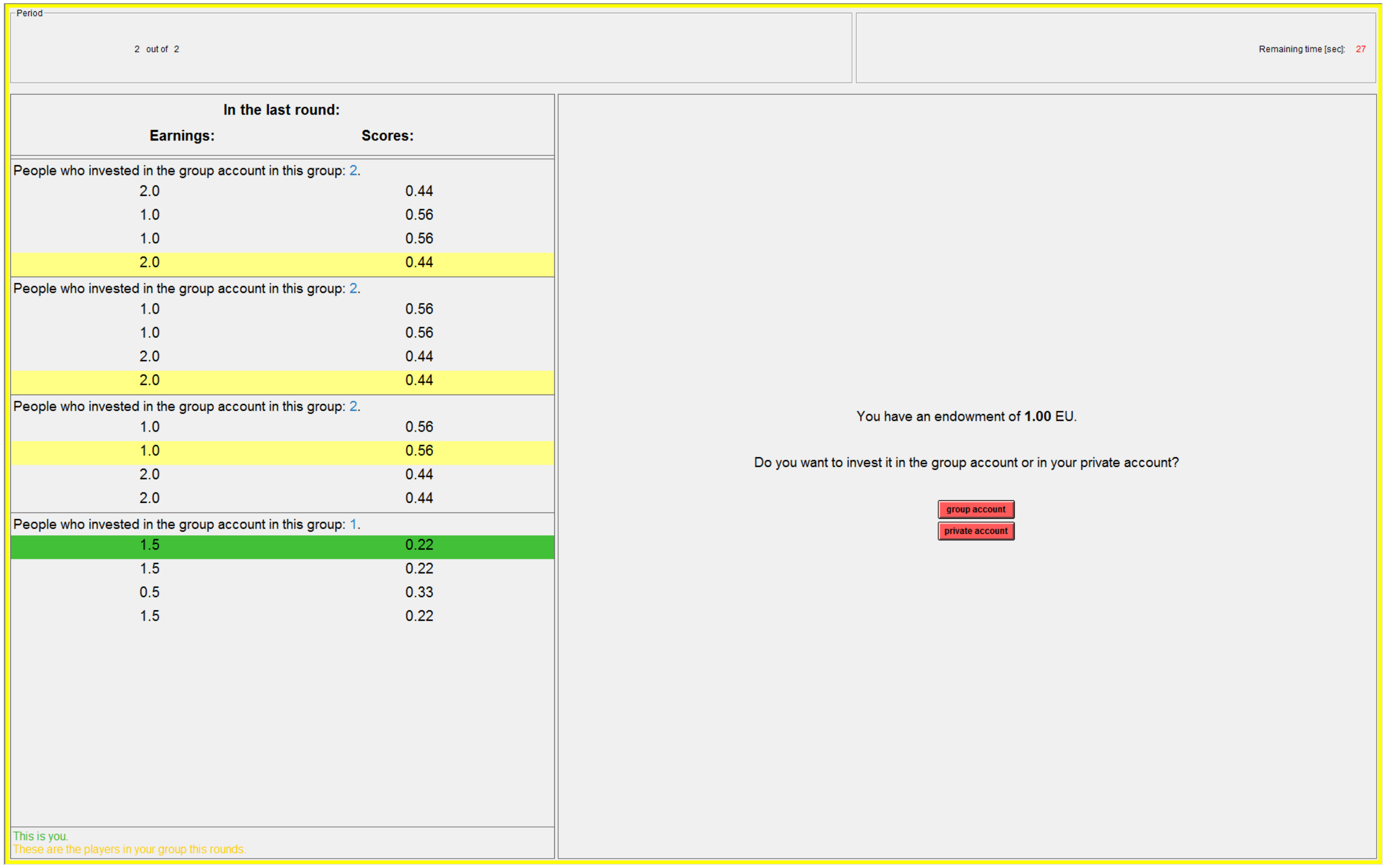

Figure S30: This is the decision screen shown to the players in the Image Self Scoring treatment (from the second round on). On the left it shows how players where grouped in the previous round and each player's (image-self) score. The green line highlights the player and the yellow lines the player's teammates in the current round.

Image self-score calculator

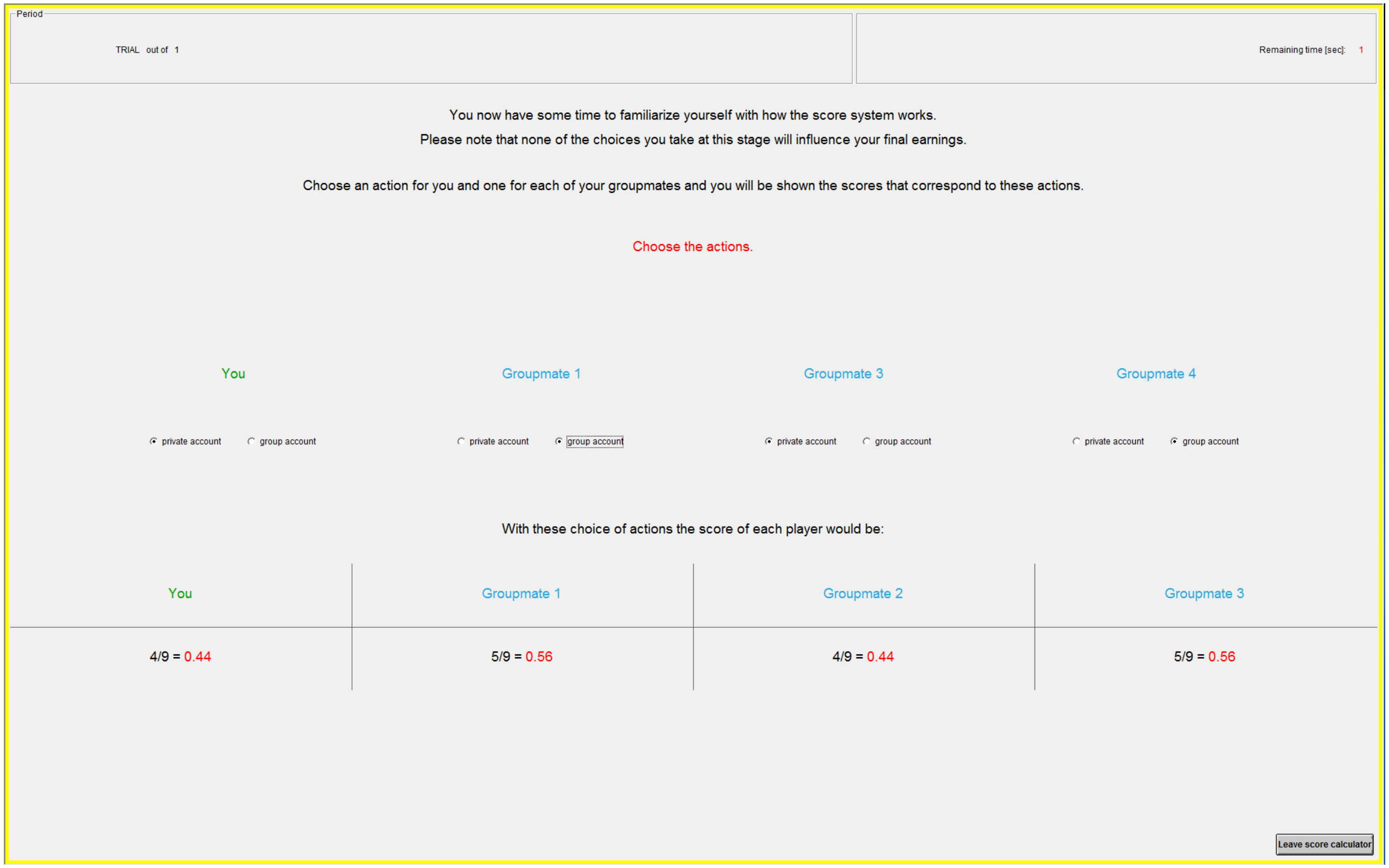

Figure S31: This is a screenshot of the Image Self score calculator. Before the Image Self score treatment, players were given time to understand the scoring mechanism by trying different combinations of theirs and their teammates actions and observe the resulting score.

S7 Dataset

All the data-points collected in the experiment are provided as a CSV file. In the following, we provide a short description of the dataset to help reproduce our findings.

The experiment featured 12 sessions with 16 participants in each session, for a total of 192 participants and 13248 individual decisions.

Each row in the database represents one decision. Its attributes are:

- ***Session:*** This is a unique identifier for each session (remember that each session consists of an Initial Phase and two Scoring Phases).
- ***Treatment:*** This indicates which treatment was currently being played: No scoring, image scoring, group scoring, truthful scoring (meaning Image Self Scoring) and self scoring.
- ***Period:*** Indicates in which of the 20 rounds in a phase the decision was taken.
- ***Subject:*** Identifies the identity of the subject in a session. Note that this uniquely identifies subjects in a session, but not in different sessions.
- ***Group:*** Indicates to which group (in that round) belonged the player that made a decision.
- ***Contributed:*** This is the decision taken by the player: 1 indicates that the player cooperated and 0 that the player did not
- ***Profit:*** This is how much profit the player made this turn.
- ***NumOfContr:*** This is how many people in the player group contributed in that round.
- ***Order:*** This indicates whether a certain decision was taken during the Initial Phase (1), the Scoring Phase 1 (2) or the Scoring Phase 2 (3).
- ***ID:*** This is a unique identifier for the Phase that the player was playing. This means that each unique sessions has three unique IDs.
- ***Image:*** This indicates (when applicable) the score of the player after that round; i.e. the result of the decision(s) in the current round that will be shown to the players in the following round.
- ***GiveStars:*** This indicates how many stars did the player assigned to his/her group in the Self Scoring treatment.
- ***PlayerIdent:*** This a unique identifier for all subjects. I.e., contrarily to the *Subject* attribute, this uniquely identifies a player in the entire dataset.

S8 Bibliography

References

[1] Brown, Lawrence D., T. Tony Cai, and Anirban DasGupta. *Interval estimation for a binomial proportion.* **Statistical science** 16.2 (2001): 101-117.

[2] Fisher, Ronald Aylmer. *The design of experiments.* Oliver And Boyd; Edinburgh; London, 1937.

[3] Good, Phillip I. *Resampling methods.* Birkhũser Boston, 2006.

[4] Collingridge, Dave S. *A primer on quantitized data analysis and permutation testing.* **Journal of Mixed Methods Research** 7.1 (2013): 81-97.

[5] Phipson, Belinda, and Gordon K. Smyth. *Permutation P-values should never be zero: calculating exact P-values when permutations are randomly drawn.* **Statistical Applications in Genetics and Molecular Biology** 9.1 (2010): 39.

[6] Fischbacher, Urs, Simon Gächter, and Ernst Fehr. *Are people conditionally cooperative? Evidence from a public goods experiment.* **Economics Letters** 71.3 (2001): 397-404.

[7] Frey, Bruno S., and Stephan Meier. *Social comparisons and pro-social behavior: testing "conditional cooperation" in a field experiment.* **American Economic Review** 94.5 (2004): 1717-1722.

[8] Keser, Claudia, and Frans Van Winden. *Conditional cooperation and voluntary contributions to public goods.* **The Scandinavian Journal of Economics** 102.1 (2000): 23-39.

[9] Philip Leifeld. *texreg: Conversion of Statistical Model Output in R to LaTeX and HTML Tables.* **Journal of Statistical Software** 55(8) (2013): 1-24.

[10] Alan Fernihough (2011). *Simple logit and probit marginal effects in R.* **Working Papers** 201122 (2011), School of Economics, University College Dublin.

[11] Andreoni, James. *Why free ride? Strategies and learning in public goods experiments.* **Journal of Public Economics** 37.3 (1988): 291-304.
