# Supplementary material for: Groups and scores: the decline of cooperation
Source: J R Soc Interface. 2018 Jul 4;15(144):20180158. doi: 10.1098/rsif.2018.0158 (PMC6073651; doi:10.1098/rsif.2018.0158)
Supplement: Instructions [file rsif20180158supp3.tar › Final_instructions/Welcome_Intro.docx]

# Welcome & General Introduction

Welcome and thank you for coming!

You are about to participate in an experimental study of human decision making.

In the next hour or so we are going to play several games and you will have the chance to earn some money.

Please pay careful attention to these instructions and the ones you will receive before each game starts.

Everybody will receive the same instructions.

Should you have any questions regarding the rules, please raise your hand and an experimenter will come to you and answer.

Communication with the other participants in the experiment is strictly forbidden.

Also, please turn off your mobile phones.

If you violate these rules we will have to exclude you from the experiment and from any earning.

You already earned 5 CHF for coming here today.

During the experiment you can earn more, depending on your decisions and on the decisions of the other players.

The currency of the experiment is the Experimental Unit (EU).

During the experiment your earnings will be calculated in EU.

At the end of the experiment your Experimental Units will be converted at a rate of 1EU = 0.33 CHF. This means for example that 60 EU will be converted into 20 CHF.

You will be paid privately, in cash, at the conclusion of the experiment.

Nobody will know your ID, please keep your ID secret.

Do not reveal it during the game in any way, and do not tell your colleagues after the game.

We are going to play a lot of interactions in the following, and it is possible that this may create a certain rhythm.

However, you do not need to follow this rhythm.

Sometimes, you might need more time to decide. Take your time.

We will play 3 different sessions. At the beginning of each session we will explain the rules of the game you are going to play.

If everything so far is clear and you have no further questions, please continue to the next set ofinstructions.
