# Supplementary material for: Groups and scores: the decline of cooperation
Source: J R Soc Interface. 2018 Jul 4;15(144):20180158. doi: 10.1098/rsif.2018.0158 (PMC6073651; doi:10.1098/rsif.2018.0158)
Supplement: Instructions [file rsif20180158supp3.tar › Final_instructions/1-2-3.docx]

## Session 1

Welcome to the first session of today!

This session will consist of 20 different rounds. In the first 3 rounds, you will have 30 seconds to make your decision and become familiar with the game. After the 3^rd^ round you will have less time. Please make sure to choose an action before the time runs out.

In each round you will be grouped with 3 other participants assigned by the computer randomly. Each round, the computer will reform the groups at random. The other participants will face the exact same task as you.

In each round, you and the other members of your group will be assigned an endowment of 1EU. You will then have to decide whether to put this amount into a shared pool belonging to your group or to keep it for you.

At the end of the round you will receive an amount of EU equal to the sum of all the contributions to the common pool multiplied by a factor of 2 shared equally among the group members. Hence, each player will receive an amount of EU equal to ½ what contained in the common pool. In addition you will receive your initial endowment, only if you decided not to invest it. Note that you will receive your share of the common pool, regardless from whether you invested your endowment or not.

At the end of each round, your round’s income will be displayed on your computer screen, together with how many people contributed to the common pool in your group. Then, a new round will start.

After 20 rounds, we will give you new instructions and a new session will begin.

If you have any questions, please raise your hand now.

Before the start of the session, you will have 2 minutes to play with a payoff calculator so that you can understand how your earnings depend on your action and on the actions of the other players.
After those 2 minutes, the experiment will start.

## Session 2

This session will also consist of 20 different rounds. In the first 3 rounds, you will have 30 seconds to make your decision and become familiar with the game. After the 3^rd^ round you will have less time. Please make sure to choose an action before the time runs out.

In this session, you are going to play a similar game to the one you played before. The only difference will be that, starting from the second round, you will be informed about the other players’ previous round actions:

A score will be assigned to each player, depending on whether or not he/she contributed to the common pool of his/her group in the previous round. A score of 1 indicates that the player did contribute last round to the common pool, while a score of 0 indicates that the player did not contribute. Note that the score is not cumulative. You will also be shown the composition of each group in the previous round.

Starting from the second round, in this session you will know who the other members of your group are. The scores from the last round of the players in your group are highlighted in yellow while your own score is highlighted in green.

At the end of each round, your round’s income will be displayed on your computer screen, together with how many people contributed to the common pool in your group. After that, a new round will start. Also in this game, each round, the computer will reform the groups at random.

After 20 rounds, we will give you new instructions and a new session will begin.

If you have any questions, please raise your hand now. The session will start once everyone has finished reading the instructions.

## Session 3

This session will also consist of 20 different rounds. In the first 3 rounds, you will have 30 seconds to make your decision and become familiar with the game. After the 3^rd^ round you will have less time. Please make sure to choose an action before the time runs out.

The game in this session is very similar to the one played in session 2. The only difference is that the score assigned to each player will not reflect his/her individual contribution but the total number of contributions in his/her group last round. This means that members of the same group will all receive the same score.

The table below shows how the number of contributions in the previous round determines the score of a player in the next round, indicating the proportion of contributors in his/her group:

| **Player X’s score** | **Number of people in X’s group contributing to the common pool.** |
| --- | --- |
| 0 | 0 |
| 0.25 | 1 |
| 0.50 | 2 |
| 0.75 | 3 |
| 1 | 4 |

At the end of each round, your round’s income will be displayed on your computer screen, together with how many people contributed to the common pool in your group. After that, a new round will start. Also in this game, each round, the computer will reform the groups at random.

Again you will know who the other members of your group are. The scores from the last round of the players in your group are highlighted in yellow while your own score is highlighted in green.

After 20 rounds, the experiment will be over. You will be shown your total earnings and be paid. Please stay seated until we ask you to come forward to collect what you have earned.

If you have any questions, please raise your hand now. The session will start once everyone has finished reading the instructions.
